# Mrc1^+^ macrophage-derived IGF1 mitigates crystal nephropathy by promoting renal tubule cell proliferation *via* the AKT/Rb signaling pathway

**DOI:** 10.7150/thno.89174

**Published:** 2024-02-17

**Authors:** Linxi Huang, Wei Chen, Zhuojing Tan, Yunxiao Huang, Xinji Gu, Lantian Liu, Hongxia Zhang, Yihan Shi, Jiarong Ding, Chengjian Zheng, Zhiyong Guo, Bing Yu

**Affiliations:** 1 Department of Cell Biology, Naval Medical University (Second Military Medical University), Shanghai, China.; 2 Department of Nephrology, Changhai Hospital, Naval Medical University (Second Military Medical University), Shanghai, China.; 3 Department of Nephrology, PLA Navy No.905 Hospital, Naval Medical University (Second Military Medical University), Shanghai, China.; 4 Department of Nephrology, Nantong Third People's Hospital, Affiliated Nantong Hospital 3 of Nantong University, Nantong 226006, Jiangsu, China.; 5 Nanjing Medical University, Nanjing, Jiangsu, China.; 6 Faculty of Pharmacy, Naval Medical University, Shanghai, China.

**Keywords:** single-cell RNA sequencing, bioinformatic analysis, crystal nephropathy, intercellular communications, macrophage, IGF1

## Abstract

**Rationale:** The present understanding of the cellular characteristics and communications in crystal nephropathy is limited. Here, molecular and cellular studies combined with single-cell RNA sequencing (scRNA-seq) were performed to investigate the changes in cell components and their interactions in glyoxylate-induced crystallized kidneys to provide promising treatments for crystal nephropathy.

**Methods:** The transcriptomes of single cells from mouse kidneys treated with glyoxylate for 0, 1, 4, or 7 days were analyzed via 10× Genomics, and the single cells were clustered and characterized by the Seurat pipeline. The potential cellular interactions between specific cell types were explored by CellChat. Molecular and cellular findings related to macrophage-to-epithelium crosstalk were validated in sodium oxalate (NaOx)-induced renal tubular epithelial cell injury in vitro and in glyoxylate-induced crystal nephropathy in vivo.

**Results:** Our established scRNA atlas of glyoxylate-induced crystalline nephropathy contained 15 cell populations with more than 40000 single cells, including relatively stable tubular cells of different segments, proliferating and injured proximal tubular cells, T cells, B cells, and myeloid and mesenchymal cells. In this study, we found that Mrc1^+^ macrophages, as a subtype of myeloid cells, increased in both the number and percentage within the myeloid population as crystal-induced injury progresses, and distinctly express IGF1, which induces the activation of a signal pathway to dominate a significant information flow towards injured and proliferating tubule cells. IGF1 promoted the repair of damaged tubular epithelial cells induced by NaOx in vitro, as well as the repair of damaged tubular epithelial cells and the recovery of disease outcomes in glyoxylate-induced nephrolithic mice in vivo.

**Conclusion:** After constructing a cellular atlas of glyoxylate-induced crystal nephropathy, we found that IGF1 derived from Mrc1^+^ macrophages attenuated crystal nephropathy through promoting renal tubule cell proliferation via the AKT/Rb signaling pathway. These findings could lead to the identification of potential therapeutic targets for the treatment of crystal nephropathy.

## Introduction

Kidney stones are mineral deposits of crystalline and organic components 1. Stone formation is highly prevalent, with rates of up to 14.8%, and still increasing further, with a recurrence rate of up to 50% within the first 5 years of the initial stone episode 2. Crystal nephropathy is an under-recognized cause of kidney disease 3. Crystals can trigger a wide range of kidney injuries that lead to acute kidney injury, chronic kidney disease, nephrolithiasis, renal colic, or nephrocalcinosis 4. Calcium oxalate constitutes approximately 80% of kidney stones 5; generally, elevated urine oxalate concentrations above the supersaturation limit caused by various conditions will lead to calcium oxalate crystallization, crystal deposition in renal tissue and finally, kidney damage evolving to renal failure 6. However, the cellular and molecular mechanisms of how crystal induces kidney tissue injury and remodeling have not yet been elucidated; specifically, there is a strong need for a detailed atlas to illustrate these processes.

The innate immune response after tubular cell damage profoundly influences the resolution or progression of crystal nephropathy. A pioneer work published decades ago corroborated the presence of macrophages and multinucleated giant cells encapsulating crystals in kidney stone patients as well as in hyperoxaluric nephrolithiasis animal models 7. Recent studies have demonstrated that macrophages are major contributors to the inflammatory response but also important mediators of tissue homeostasis and host defense against kidney injury 8. Depending on the complex signals in the damaged microenvironment, macrophages can differentiate into a dynamic and diverse spectrum of phenotypes and functional states. Nevertheless, our understanding of macrophage origins, functions, and activation regulations at organ and cellular levels is largely limited 9-11.

Single-cell RNA sequencing (scRNA-seq) has emerged as a powerful tool for unbiasedly revealing cellular heterogeneities, identities, and states independent of a *priori-*defined labeling strategies 12, 13. Recent advances in scRNA-seq have facilitated detailed analysis of kidney cell molecular definitions, kidney cell composition changes, and immune cell responses across species in multiple kidney diseases 14-17. However, the cellular heterogeneity and immune responses in crystal nephropathy remain unclear. Herein, we aimed to construct a cellular atlas of crystal nephropathy utilizing a glyoxylate-induced hyperoxaluric mouse model system 18. We observed time-course changes of injured as well as proliferating tubules after crystal damage. Consistent with the findings of previous studies, our findings further revealed the infiltration of immune cells, including myeloid cells, in diverse states 16, 19, 20. Moreover, we identified a subset of macrophages, labeled with the canonical M2 macrophage marker Mrc1, that distinctly expressed insulin-like the growth factor 1 (IGF1). We subsequently evaluated the effect of IGF1 on crystal-induced injury in tubular epithelial cells in vivo and in vitro. Our results revealed cellular diversity, particularly among macrophages, after crystal damage and suggested precise therapeutic targets for treating crystal nephropathy.

## Methods

### Mouse models and single-cell RNA sequencing

Male C57BL/6 mice aged 8 weeks were purchased from Shanghai Lingchang Biotechnology Co., Ltd., and were reared under standard pathogen-free conditions with ad libitum access to water and food. All animal experiments were approved by the Committee on Ethics of Biomedicine, Second Military Medical University. The calcium oxalate (CaOx) crystal nephropathy model was established by intraperitoneal injection of glyoxylate (Gly, 80 mg/kg/d; Tokyo Chemical Industry Co. Ltd.) for 0, 1, 4 and 7 successive days 18. Kidneys were harvested on the indicated days for scRNA-seq and histologic verification (n=3 per group). scRNA-seq was performed according to the protocols of 10× Genomics, and the detailed process of the data analysis is presented in the Supplementary methods. Crystallized mice were supplemented with recombinant human IGF1 (100-11, PeproTech) subcutaneously for the indicated days (n=6 per group). Before the establishment of the Gly-induced crystal nephropathy model, each mouse was intravenously injected with a single dose of 100 μg of the IGF1 antibody (MAB791, R&D) and was sacrificed on Day 4 to collect kidney samples 21.

### Bone marrow-derived macrophages

Bone marrow-derived macrophages (BMDMs) were isolated from the femurs of C57BL/6 mice. Mononuclear cells flushed from the bone marrow were maintained in Dulbecco's modified Eagle's medium (DMEM; Corning) supplemented with 10% fetal bovine serum (ZQ100; Shanghai Zhong Qiao Xin Zhou Biotechnology) and 20 ng/ml colony stimulating factor 1 (CSF-1; PeproTech) for 1 week to obtain BMDMs.

### Cell culture and conditioned medium

Mouse renal tubule epithelial cells (mRTECs) were cultured in DMEM supplemented with 10% FBS and 100 U/ml penicillin‒streptomycin. Human proximal tubular HK-2 cells were cultured in DMEM-F12 (HyClone) supplemented with 10% FBS and 100 U/ml penicillin‒streptomycin according to previous methods 22. mRTECs and HK-2 cells were separately cultured to prepare the conditioned media. Briefly, cultured mRTECs or HK-2 cells were separately challenged with or without 1.72 mM and 0.5 mM NaOx for 24 h. Next, the culture media was collected and centrifuged to obtain the supernatants. Then, the supernatants were collected as conditioned media to stimulate BMDMs. Media containing 1.72 mM or 0.5 mM NaOx without mRTECs or HK-2 cells were incubated for 24 h and were used as controls.

### Online data source

The GSE174324 dataset contains single-cell sequencing data on mononuclear phagocytes in the kidneys, blood and spleen of mice after unilateral ischemia-reperfusion injury (IRI). Renal cell data were downloaded and reprocessed based on the authors' description 16.

### Immunohistochemistry (IHC), immunofluorescence (IF), western blotting and real-time PCR

IHC, IF, western blot, and real-time PCR were performed as described previously 23, 24. The detailed procedures are presented in the Supplementary methods.

### Cell viability

A CCK8 assay kit (Dojindo, CK04) was used to detect cell viability according to the manufacturer's procedure. The absorbance of each well was measured at a wavelength of 450 nm by a Molecular Devices Spectramax 190 microplate reader. Cell survival rates are shown as a percentage of the absorbance of the control cells.

### EdU assay

EdU staining was conducted following the manufacturer's instructions (Beyotime, China). Briefly, the cells were incubated with 1× EdU working solution at 37 °C for 4 h, followed by fixation in 4% formaldehyde. The fixed cells were labeled with a click reaction cocktail, and the nuclei were counterstained with Hoechst 33342. The samples were imaged using Nikon Eclipse Ti2 fluorescence microscopy.

### Flow cytometry assay

To detect the expression levels of MRC1 (encoding protein CD206) and IGF1 in BMDMs, the treated cells were harvested and washed twice with phosphate-buffered saline (PBS). Then, the cells were fixed with 4% paraformaldehyde (PFA) for 30 min at 4 °C and permeabilized for 10 min in staining buffer. The cells were incubated with an anti-IGF1 antibody (ab9572) for 30 min. Then, the cells were washed twice and incubated with anti-rabbit IgG-Cy5 and FITC-conjugated anti-mouse CD206 (BioLegend, 141703) for 30 min. The stained cells were washed twice and analyzed with a Beckman Coulter CytoFLEX flow cytometry system. The fixed and permeabilized cells were incubated with anti-rabbit IgG-Cy5, and FITC-rat IgG2a (κ Isotype) control antibody (BioLegend, 400505) as negative controls.

To determine the cell cycle distribution in living mRTECs and HK-2 cells, the cells were dissociated into single cells by trypsinization and harvested by centrifugation at 200 × g for 5 min. The cells were resuspended in PBS and incubated with Zombie Green™ dye (BioLegend, 423111) in the dark for 30 min at room temperature. After one wash with 2 ml of PBS containing 10% FBS, the cells were fixed with paraformaldehyde. After three washes with PBS, the cells were stained with 50 µg/ml propidium iodide (PI) containing 20 µg/ml RNase A. Flow cytometry analyses were performed with FITC and PE channels. The data were analyzed by FlowJo V10 software.

### Statistical analysis

The data are presented as the mean ± standard deviation (SD). Statistical analysis was conducted using GraphPad Prism 9 software, the statistical methods are indicated in the figure legends, and *p* < 0.05 was considered statistically significant.

## Results

### Single-cell transcriptomic profiling of oxalate insult-insulted mouse kidneys

**Figure 1:**
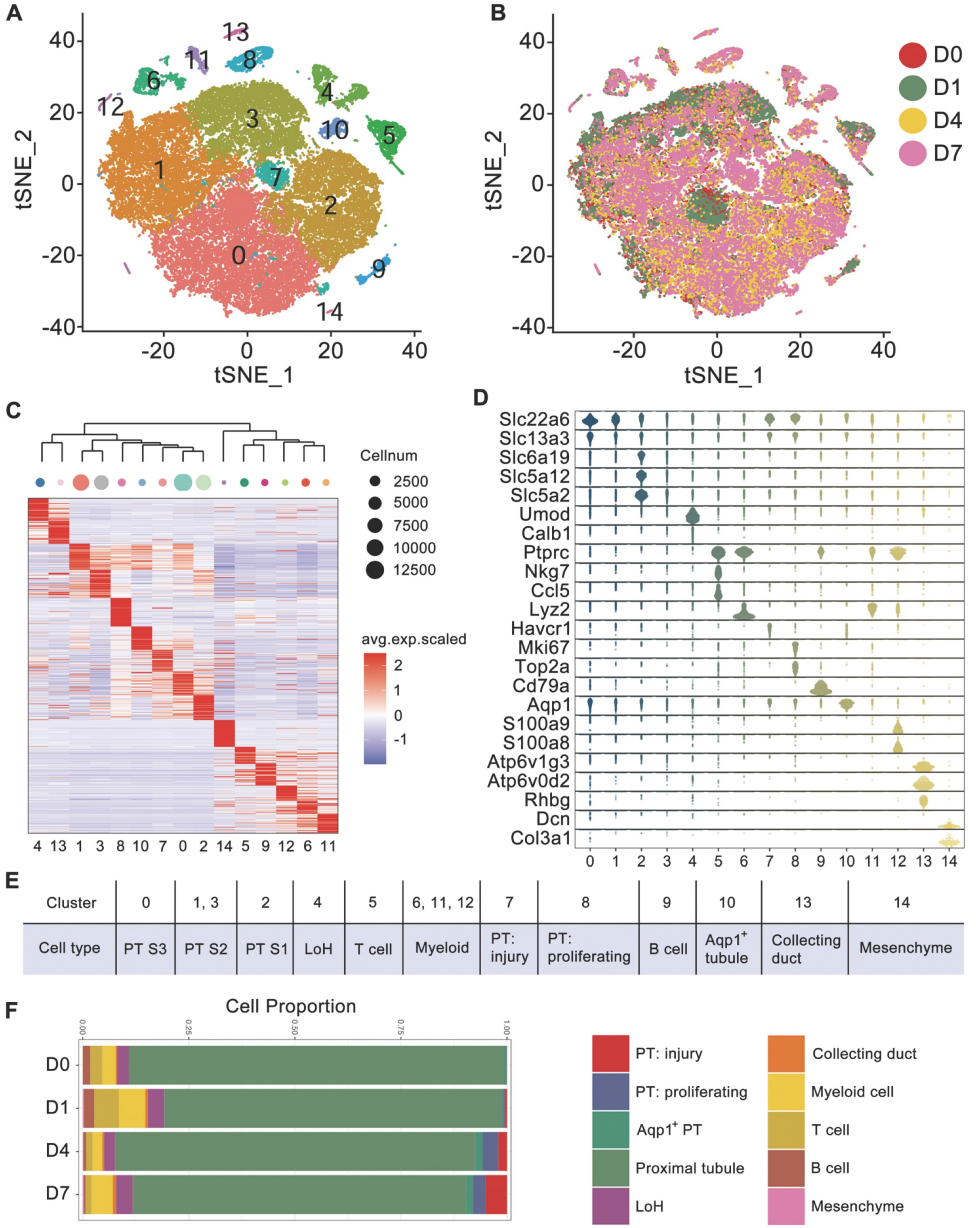
** Cell diversity in glyoxylate-injured mouse kidneys delineated by single-cell transcriptomic analysis**. **A** Unsupervised clustering of 15 cell clusters in a tSNE map. **B** tSNE map of mouse kidney cells, color coded according to the experimental groups. **C** Heatmap showing the differentially expressed genes in each cluster. Based on the expression pattern of each cluster, the clusters were clustered according to their distance calculated by the Euclidean algorithm. **D** Violin plots showing the expression levels of representative marker genes across the 15 main clusters. The y-axis is the log scale-normalized read count. **E** Cell type annotation of different clusters. **F** The percentage change in specific cell types across different experiments.

To dissect the cellular heterogeneity and to explore the key changes in injured kidney caused by crystals, we performed droplet-based single-cell RNA sequencing on kidney cells collected from mice receiving glyoxylate treatment for 0, 1, 4 or 7 consecutive days. After stringent quality control and filtering, 45350 transcriptomes of single cells were obtained (Figure S1) and were clustered into 15 clusters by the t-distributed stochastic neighbor embedding (tSNE) reduction (Figure 1A). The transcriptomes of cells obtained at different times exhibited weak batch effects (Figure 1B). Differential gene expression analysis identified cluster-specific marker genes (Figure 1C and Table S1). Combined with differentially expressed genes (DEGs), several known kidney cell type-specific markers were utilized to annotate cell populations (Figure 1D).

Both Slc22a6 and Slc13a3 are canonical markers for proximal tubule (PT) cells, Umod is a marker for loop of Henle (LoH) cells, Ptprc is a marker for immune cells, both Ccl5 and Nkg7 are markers for T cells, Cd79a is a marker for B cells, Lyz2 is a marker for myeloid cells, and Atp6v1g3 is a marker for collecting duct cells. Cluster 14 included a mere 100 cells and exclusively expressed Dcn and Col3a1; these cells were identified as mesenchymal cells. Intriguingly, we observed that the injury marker Havcr1 and the proliferation markers Mki67 and Top2a were exclusively expressed in Clusters 7 and 8, which also expressed PT markers. These cells were subsequently identified as injured and proliferating PT cells, respectively.

To further distinguish each tubular segment, the segment markers and the DEGs referenced from published scRNA-seq data were utilized 14, 25, and the expression patterns of the PT clusters were distinguished. The results indicated that Slc6a19, Slc5a12 and Slc5a2, which were reported as S1 PT markers 25, were exclusively expressed in Cluster 2 (Figure 1D). In addition, the results from the heatmap of DEGs also indicated that Cluster 2 was closer to the proximal S1 segment, Cluster 0 was closer to the proximal S3 segment, and Clusters 1 and 3 were closer to the S2 segment. The heatmap also indicated that injured and proliferating tubules were more closely related to the S2 and S3 proximal segments (Figure S2A). Finally, the 15 clusters were annotated as 12 distinct cell types (Figure 1E). Moreover, both the percentage of injured and proliferating PT cells increased after glyoxylate treatment (Figure 1F). Trajectory analysis of kidney epithelial cells, including PT cells, LoH cells, and collecting duct cells, indicated the transition of PT cells to a proliferative state after crystal damage (Figure S2B-C).

### Myeloid subsets with distinct functions are identified during kidney injury

**Figure 2:**
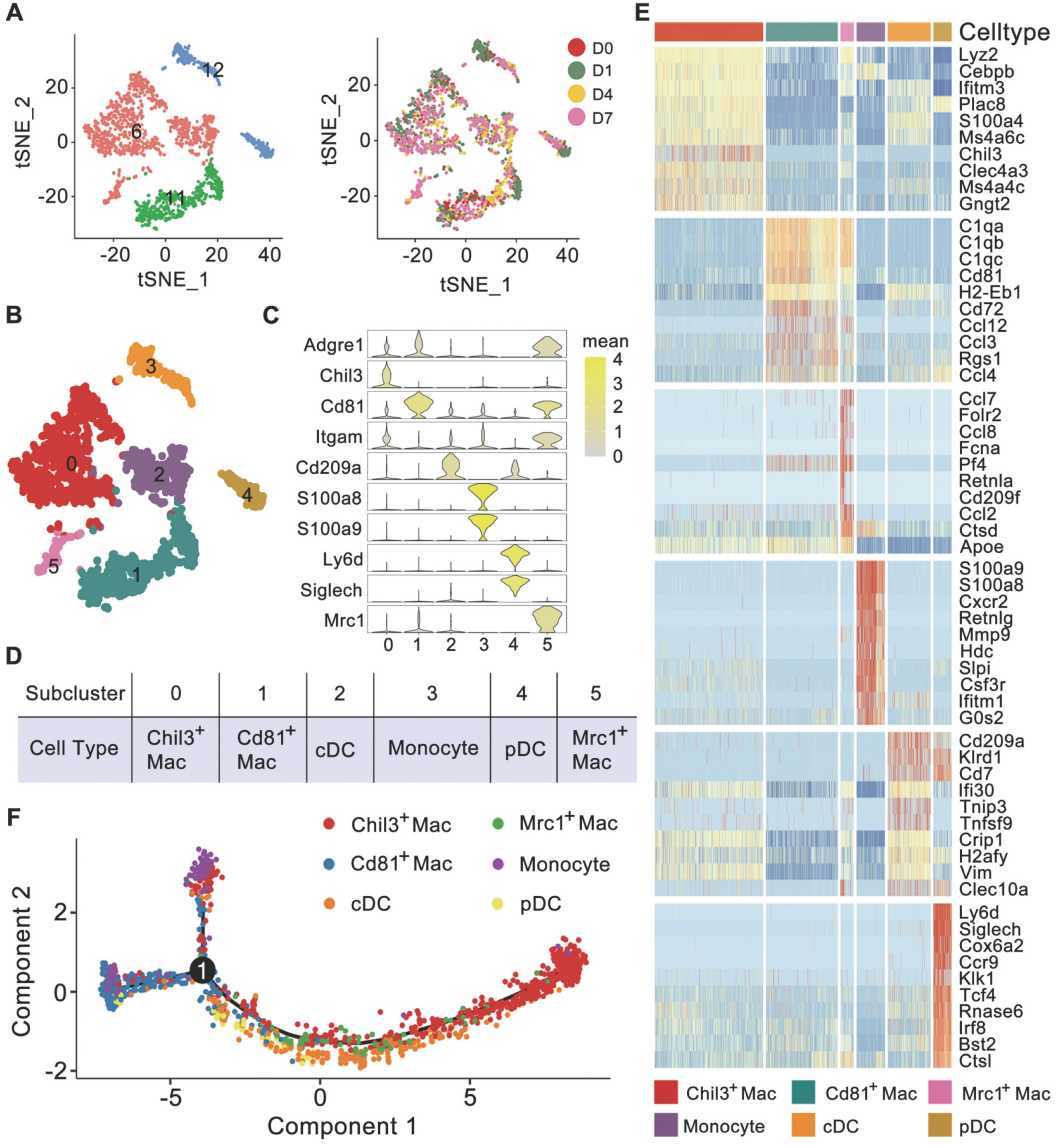
** Cell diversity of myeloid cells in glyoxylate-insulted mouse kidneys**. **A** Left: tSNE map of myeloid cells, which consisted of Clusters 6, 11 and 12 identified at primary clustering. Right: tSNE map colored according to the experimental groups. **B** Unsupervised sub-clustering of myeloid cells revealed 6 cell subclusters in the tSNE map. **C** Violin plots showing the expression levels of representative marker genes across the 6 myeloid cell subclusters. The X-axis shows the log scale-normalized read count. **D** Cell type annotation of distinct subtypes of myeloid cells. **E** Heatmap showing genes elevated in different myeloid cell populations relative to each other based on the z score. Only the top 10 differentially expressed genes ranked by the average logFC of expression in each cluster are shown. **F** Trajectory analysis of different myeloid cell types suggesting distinct gene expression features in Chil3^+^ macrophages and Cd81^+^ macrophages. The Mrc1^+^ macrophages were more similar to Chil3^+^ macrophages according to the single-cell mRNA expression patterns. Number 1 represents the significant branch points of differentiation.

Myeloid cells are important immune cells that reside in healthy kidneys or invade injured kidneys. Based on the high Lyz2 expression, we first identified myeloid cells composed of primitive 6, 11, and 12 clusters (Figure 1D-E), and no batch effects were detected among the 4 experiments (Figure 2A). Further clustering analysis identified 6 subclusters (Figure 2B and Table S2). Subclusters 0, 1 and 5 expressed high-levels of canonical macrophage marker Adgre1, subcluster 0 exclusively expressed Chil3, subcluster 1 expressed Cd81, and subcluster 5 expressed Mrc1 (encoding Cd206). Subcluster 3 exclusively expressed the conventional monocyte markers S100a8 and S100a9. Subclusters 2 and 4 exclusively expressed the dendritic cell (DC) marker Cd209a, while subcluster 4 also exclusively expressed the plasmacytoid DC (pDC) markers Ly6d and Siglech (Figure 2C). A heatmap of DC marker genes identified in another single-cell analysis of mouse kidney mononuclear phagocytic cells revealed similar expression patterns between conventional DCs (cDCs) and subcluster 2 and between pDCs and subcluster 4 (Figure S3) 16. The relatively higher expression level of Itgam in subclusters 0, 2, 3 and 5 suggested that their origination as infiltrating. Accordingly, we annotated these 6 myeloid subclusters as Chil3^+^ macrophages, Cd81^+^ macrophages, Mrc1^+^ macrophages, monocytes, cDCs and pDCs (Figure 2D). Moreover, these cells scarcely expressed fibrosis markers, such as Ankrd1, Dcn, Mgp and Ptgds, indicating that these cells were not in pro-fibrotic phenotype (Figure S4A). The heatmap indicated that each sub-cluster had a discrete transcriptional profile (Figure 2E). Trajectory analysis clearly separated Chil3^+^ macrophages from Cd81^+^ macrophages, whereas Mrc1^+^ macrophages showed a transcript profile similar to that of Chil3^+^ macrophages (Figure 2F).

**Figure 3:**
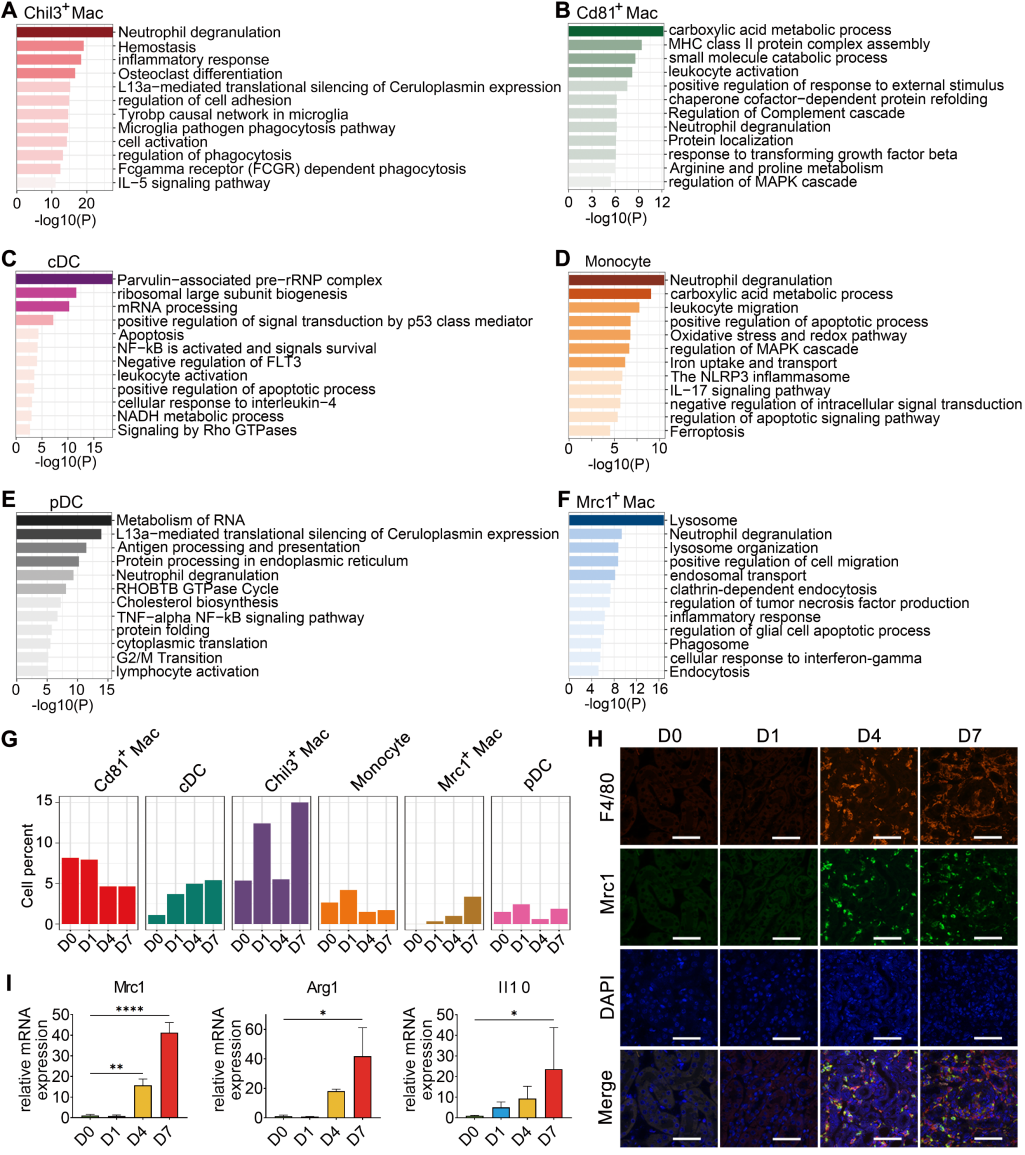
**Cellular changes and functions of myeloid cell subtypes after glyoxylate treatment**.** A-F** Biological functions and pathways enriched from marker genes of different myeloid cell subtypes, including Chil3^+^ macrophages (A), Cd81^+^ macrophages (B), cDCs (C), monocytes (D), pDCs (E) and Mrc1^+^ macrophages (F), annotated by Metascape. **G** The percentage change of specific myeloid cell types across different experiments. **H** Immunofluorescence staining of Mrc1 and F4/80 in kidneys glyoxylate-treated on D0, 1, 4 and 7 validates the recruitment and generation of Mrc1^+^ macrophages (arrow). Scale bar: 50 µm.** I** Quantitative PCR showing the fold changes in Mrc1, Arg1 and Il10 expression across the experiments. One-way ANOVA analysis. *, p < 0.05, ** < 0.01, *** < 0.001.

To gain further insights into the characteristics and roles of the identified myeloid subclusters, Metascape was used to construct functional enrichment analysis and protein-protein interaction networks of DEGs from specific myeloid subsets. Chil3^+^ macrophages, usually recognized as canonical M2 macrophages 26, were mainly engaged in phagocytosis (Figure 3A and Figure S4B). Cd81^+^ macrophages, commonly recognized as kidney resident macrophages 17, were mainly involved in MHC class II protein complex assembly and antigen processing and presentation (Figure 3B and Figure S4B), which is consistent with previously identified CD81^+^ resident macrophages in rat and human kidneys 27. cDCs were engaged in the cellular response to interleukin-4, suggesting their involvement in the inflammatory response after crystal-induced injury (Figure 3C). Monocytes contributed to the NLRP3 inflammasome pathway (Figure 3D), while pDCs acted in antigen processing and presentation (Figure 3E and Figure S4B). Mrc1^+^ macrophages engaged in the phagosome formation, endocytosis and responding to interferon-gamma (Figure 3F). These activities suggest that Mrc1^+^ macrophages possess immunosuppressive characteristics, which play a role in preventing an exacerbated inflammatory response after injury 28. The 6 myeloid cell subsets exhibited dynamic changes during disease progression. Notably, the percentage of Mrc1^+^ macrophages increased after glyoxylate treatment (Figure 3G-H). Quantification of the expression of the canonical M2 macrophage markers Mrc1, Arg1 and Il10 revealed consistent upregulation (Figure 3I and Figure S5). These results strongly indicate that anti-inflammatory macrophage populations are recruited to the kidneys after crystal-induced injury.

### Strengthened IGF signaling in Mrc1^+^ macrophages facilitates renal self-repair after crystal-induced injury

**Figure 4:**
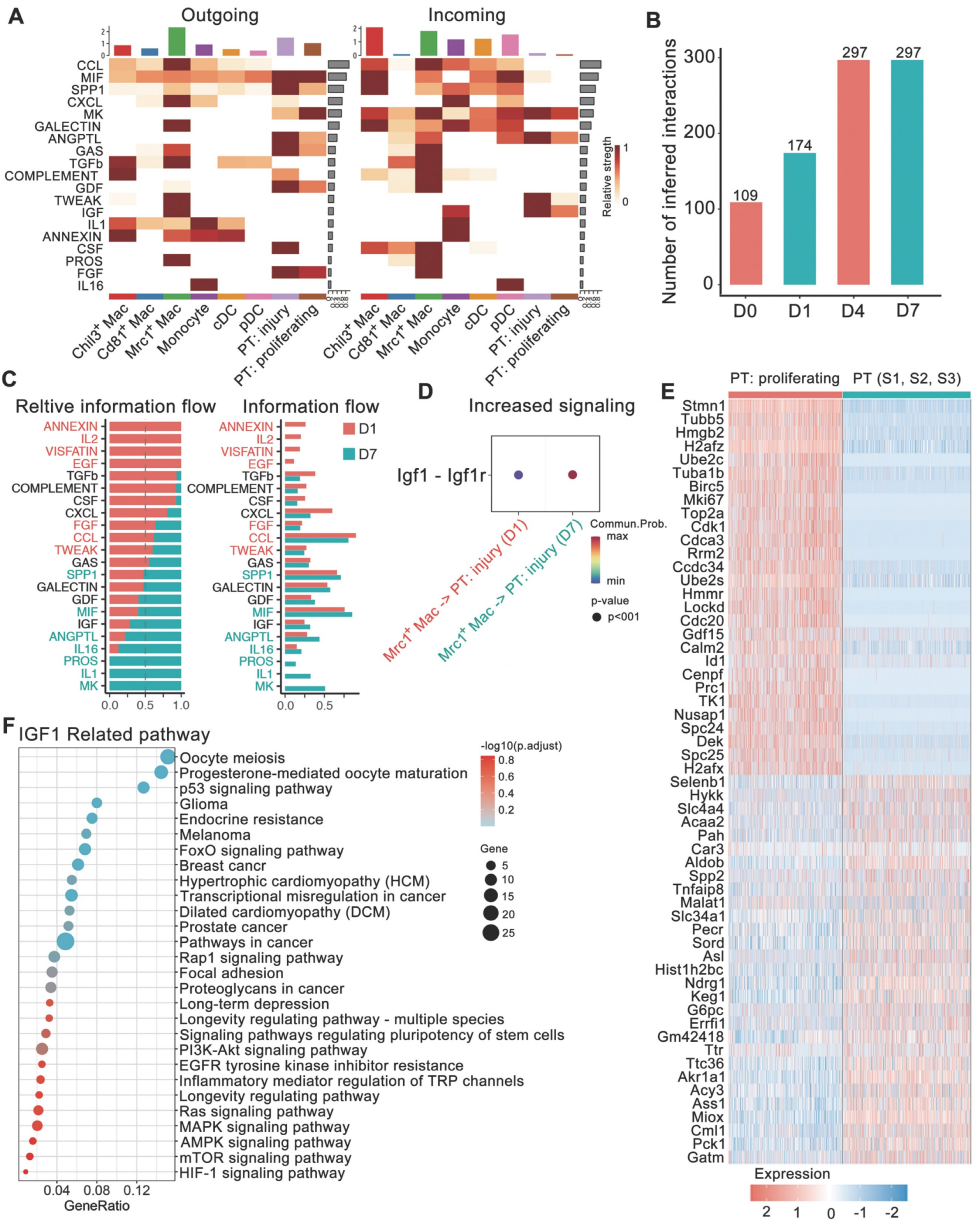
** Changes in ligand-receptor interactions between myeloid cells and 2 dynamic tubule cell types with disease progression. A** Heatmaps summarizing specific signals between interacting cell types. Interactions are divided into outgoing and incoming events for specific cell types. The color gradient indicates the relative strength of the interactions. **B** Bar plots displaying the changes in the number of interactions between cell types across the experiments. **C** All significant signaling pathways were ranked based on their differences in overall information flow within the inferred networks between glyoxylate-treated D1 and D7 kidneys. The overall information flow of a signaling network was calculated by summing all the communication probabilities in that network. **D** Comparison of the significant ligand‒receptor signals directed from Mrc1^+^ macrophages to injured tubule cells between D1 and D7 kidneys indicated increased Igf1 signaling. The dot color reflects the communication probabilities, and the dot size represents the computed p value. **E** Heatmap showing the distinct expression patterns of proliferating tubule cells compared to proximal tubule cells. F IGF1-related signals are widely enriched in marker genes of proliferating proximal tubule cells.

CellChat was utilized to predict putative interactions between myeloid subtypes and dynamic PT cells, including injured and proliferating PT cells 29. A total of 518 significant ligand-receptor pairs were found among the 8 cell types and further categorized into 19 signaling pathways, namely, the CCL, MIF, SPP1, CXCL, MK, GALECITN, ANGPTL, GAS, TGFb, COMPLEMENT, GDF, TWEAK, IGF, IL1, ANNEXIN, CSF, PROS, FGF and IL16 pathways (Figure S6A-B). To explore the detailed communication of individual pathways, we conducted a network centrality analysis visualized through a heatmap showing the relative importance of signals between different cell types (Figure 4A). For example, MIF and SPP1 signals, which are composed of ligand-receptor pairs such as MIf-Cd74^+^ Cxcr4 or Cd44 and Spp1-Cd44 or Itga4^+^ Itgb1, constituted the predominant information sent from PT cells to myeloid cells (Figure 4A and Figure S6C). Simultaneously, myeloid cells dominated 3 signals to PT cells, including TWEAK, MIF, and IGF1 signals. The contributions of the ligand-receptor pairs indicated the contributions of Chil3^+^ Mac and Mrc1^+^ Mac cells to the secretion of the ligand Tnfsf12 and all myeloid cell types to the ligand Mif. Notably, Mrc1^+^ macrophages distinctly expressed Igf1, dominating information to injured and proliferating PT cells (Figure S6D).

We also compared the information flow across different time points. The sum of the number of communications increased from D0 to D7 (Figure 4B). The flow of information in the TWEAK pathway (red in Figure 4C) prominently decreased at D7 compared to D1, while the flow of information in the IGF1 pathway increased (Figure 4C-D). The generation of proliferative PT cells suggested the important self-repair progression after injury. TWEAK and IGF1 signals both dominated information towards proliferative PTs. However, KEGG pathway enrichment of upregulated genes in proliferative PTs versus relatively stable PT cells (including S1, 2 and 3 segments) indicated the involvement of these genes in IGF1-related pathways, including the MAPK signaling, P53 signaling, PI3K-Akt signaling and signaling pathways regulating the pluripotency of stem cells (Figure 4E-F). IGF signaling from Mrc1^+^ macrophages to tubule cells is likely to boost cell growth. The above results suggest the important role of Mrc1^+^ macrophages in renal self-repair after crystal injury through strengthening IGF signaling.

### The IGF1-expressing macrophage subset is conserved after kidney injury

**Figure 5:**
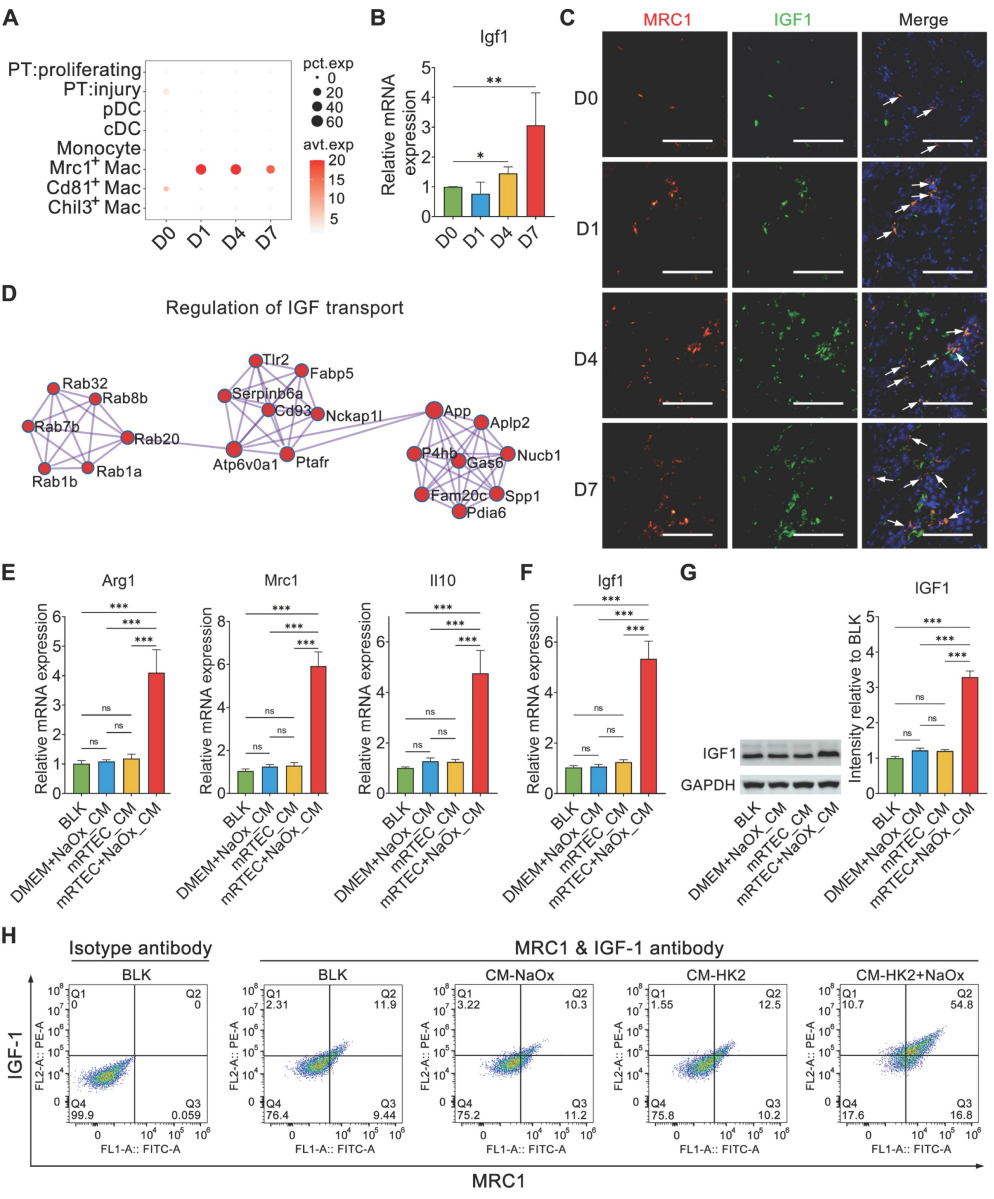
** Activation of Mrc1^+^ macrophages increased the expression of IGF1**. **A** Dotplot showing the changes in Igf1 expression in myeloid cells and proliferating and injured PT cells across the experiments. **B** The results of quantitative PCR showing the fold change in Igf1 expression in mouse kidneys after glyoxylate treatment. One-way ANOVA analysis. *, p < 0.05, ** < 0.01. **C** Immunofluorescence staining showing the change in Igf1 expression (green) in Mrc1^+^ macrophages (red) across the experiments. Arrows indicate both Mrc1- and Igf1-positive macrophages. Scale bar: 50 µm.** D** The top protein‒protein interaction network constructed via marker genes of Mrc1^+^ macrophages identified by the MCODE algorithm indicating the involvement of these genes in IGF transport regulation. **E** The results of quantitative PCR showing the fold changes in Arg1, Mrc1 and Il10 expression in BMDMs treated with conditioned medium prepared from mRTECs with or without NaOx insult. Two-way ANOVA analysis. *** < 0.001; ns, not significant. **F** The results of quantitative PCR showing changes in Igf1 expression in BMDMs. Two-way ANOVA analysis. *** < 0.001; ns, not significant. **G**. The results of western blot staining showing that the IGF1 abundance changes in BMDMs. Two-way ANOVA analysis. *** < 0.001; ns, not significant. **H** Flow cytometry showing that stimulation with NaOX-conditioned medium increased the Mrc1 and IGF1 double-positive cell ratio from 12.5% to 54.8%.

Single-cell RNA sequencing revealed that Igf1 was expressed mainly in Mrc1^+^ macrophages throughout the whole process of kidney crystal injury (Figure 5A and Figure S7). qPCR results confirmed that Igf1 was upregulated in the crystallized kidneys (Figure 5B). IF staining confirmed that IGF1 was co-localized with MRC1 in the crystalized kidney (Figure 5C). PPI analysis of the DEGs of Mrc1^+^ macrophages indicated that a network was involved in the regulation of insulin-like growth factor (IGF) transport (Figure 5D), indicating that Mrc1^+^ macrophages are equipped with IGF1 production and secretion.

We further verified the features of the Mrc1^+^ Igf1^+^ macrophage subset in a unilateral ischemia-reperfusion injury (IRI) model (GSE174324). The cells collected at D0, 1 and 3 after IRI were divided into 10 clusters (Figure S8A-B). Vlnplots indicated that Cluster 2 expressed high levels of Mrc1 and exclusively expressed Igf1 (Figure S8C). Feature plots also showed elevated expression of Mrc1 and Igf1 in Cluster 2 (Figure S8D-E). The percentage of cells in Cluster 2, Mrc1^+^ Igf1^+^ macrophages, increased sharply with disease progression (Figure S8F). Using the top 30 upregulated DEGs of Mrc1^+^ macrophages identified in our scRNA-seq data, we scored these mononuclear cells, and Cluster 2 was scored as distinctly high (Figure S8G). These results suggested that the recruitment and generation of Mrc1^+^ Igf1^+^ macrophages can be a common and important protective mechanism involved in the innate immune response after renal injury.

To verify that the injured renal epithelium contributes to shaping the macrophage phenotype characterized by IGF1 production, BMDMs were treated with conditioned medium from mRTECs treated with or without NaOx. The expression levels of Arg1, Mrc1 and Il10 in BMDMs were significantly increased in the NaOx-treated group (Figure 5E), and this change was accompanied by IGF1 upregulation (Figure 5F-G). Flow cytometry further confirmed that the conditioned medium prepared from the culture of oxalate-stimulated mRTECs enhanced the Mrc1^+^ phenotype of the analyzed macrophages and increased their IGF1 expression (Figure 5H). Moreover, the conditioned media prepared from cultured NaOx-challenged human HK2 cells were found to have similar effects on promoting IGF1 expression in BMDMs and enhancing the Mrc1^+^ phenotype (Figure S9). These results suggested that the crystal-injured renal tubular epithelium participates in shaping Mrc1^+^ macrophages.

### IGF1 alleviates kidney injury caused by crystals by facilitating the proliferation of tubular cells

**Figure 6:**
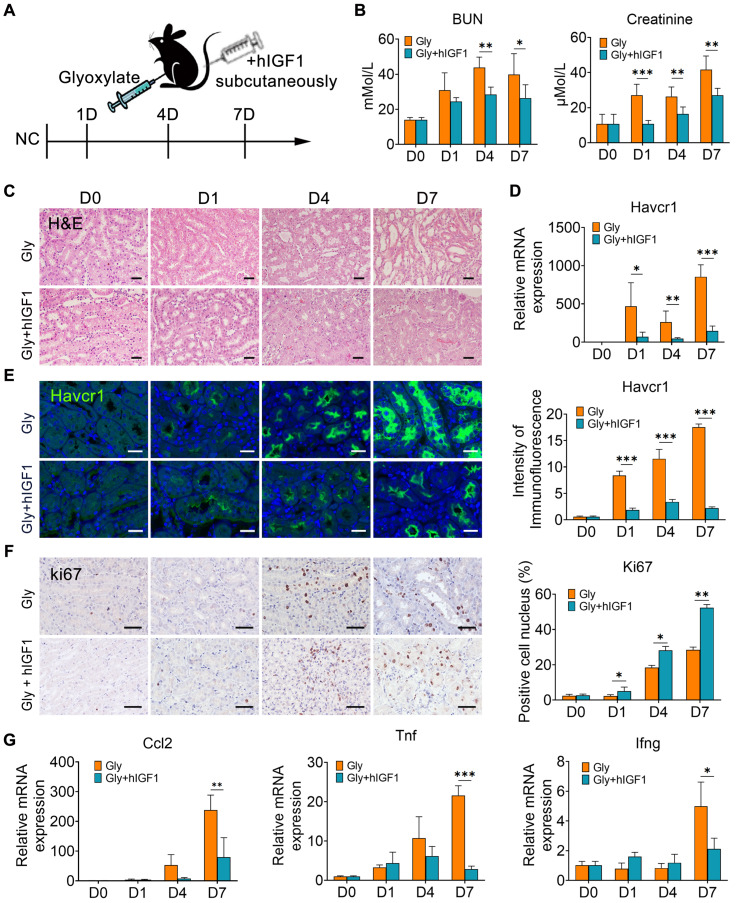
** IGF1 alleviates glyoxylate-induced kidney injury**. **A** Schematic diagram of the experimental design. One dose of IGF1 was administered to the glyoxylate-treated mice for consecutive treatments at Days 0, 1, 4 or 7. **B** The serum BUN and creatinine levels decreased after IGF1 administration to glyoxylate-induced kidney injury mice. **C** Representative results of the HE staining assay for the different groups of mice. Glyoxylate induces apparent tubular atrophy, lumen dilation, and interstitial inflammation with an increase in nucleated cell infiltration, but IGF1 attenuates these morphological changes. **D** The results of quantitative PCR showing the fold change in Havcr1 expression in mouse kidneys after glyoxylate treatment. **E** Immunofluorescence staining showing the spatial distribution and changes in Havcr1 expression (green). **F** Representative results presented with photomicrographs of immunohistochemical staining for Ki67 indicating that glyoxylate induces an increase in tubular proliferation and that IGF1 further promotes this increase. **G** The results of quantitative PCR showing the fold changes in Ccl2, Tnf and Ifng expression. Student's t test, * *p* < 0.05, ** *p* < 0.01, *** *p* < 0.001; Scale bar: 50 µm.

To verify the role of IGF1 in promoting the repair of crystallized kidneys, mice were supplemented with human recombinant IGF1 after glyoxylate treatment for the indicated days (Figure 6A). Compared with those in the crystallized group, IGF1 administration significantly reduced the increase in the serum BUN and creatinine levels caused by crystal injury (Figure 6B). The results of the HE staining assay indicated apparent tubular atrophy, especially reflected by dilated lumens, and increased nucleated cell infiltration in the glyoxylate-treated groups. On the other hand, IGF1 attenuated these morphological changes (Figure 6C). The expression of Havcr1, a specific marker of tubular cell damage, was significantly downregulated after IGF1 administration (Figure 6D-E). Moreover, the injured tubule cells positive for Havcr1 specifically expressed IGF1 receptors (Figure S10), suggesting that they were the target of IGF1 activation after ligand-receptor binding. Moreover, IGF1 treatment increased the number of Ki67-positive tubular epithelial cells (Figure 6F), especially the proportion of Ki67-positive cells among Havcr1-positive tubular cells (Figure S11), indicating that IGF1 promoted the proliferation of injured tubular cells.

**Figure 7:**
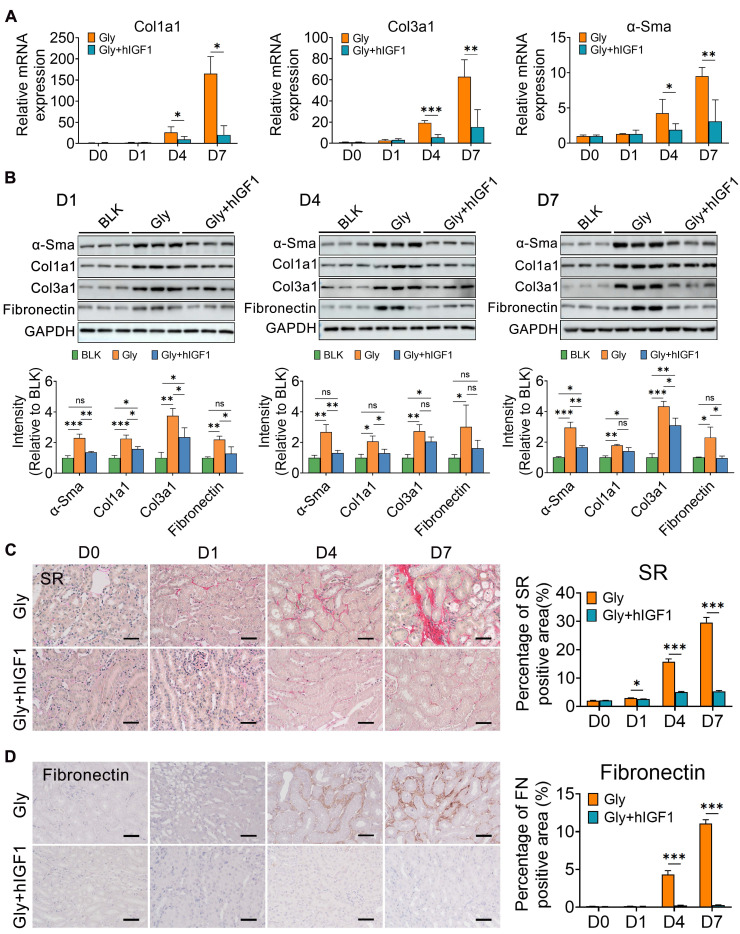
** IGF1 alleviates the fibrogenesis in glyoxylate-induced kidney**. **A** The results of quantitative PCR showing the fold changes in Col1α1, Col3α1 and α-SMA expression. **B** Western blot staining showing changes in the expression of α-SMA, Col1α1, Col3α1, and fibronectin across the experiments. **C-D** Representative results are presented with photomicrographs of kidney tissues stained with Sirius red (C) and fibronectin (D), which indicate that the progression of renal fibrosis can be blocked by IGF1 treatment. Student's t test, * *p* < 0.05, ** *p* < 0.01, *** *p* < 0.001; Scale bar: 50 µm.

Simultaneously, the number of macrophages, including Mrc1^+^ macrophages, decreased in the mice treated with IGF1 (Figure S12). These results may be due to the recovery of epithelial structure with decreasing induction of chemokines and cytokines, such as Ccl2, Tnf and Ifng (Figure 6G). In addition, IGF1 administration effectively suppressed the early fibrotic response by downregulating the expression of fibrotic genes and proteins, such as Cola1, Col3a1 and α-Sma (Figure 7A-B). Sirius red and IHC staining showed that IGF1 reduced the deposition of collagens and fibronectin in the injured kidney (Figure 7C-D). The results emphasized the importance of the proliferation and restoration of tubule cells after injury, which also play roles in subsequent inflammatory and fibrotic responses 30, 31.

**Figure 8:**
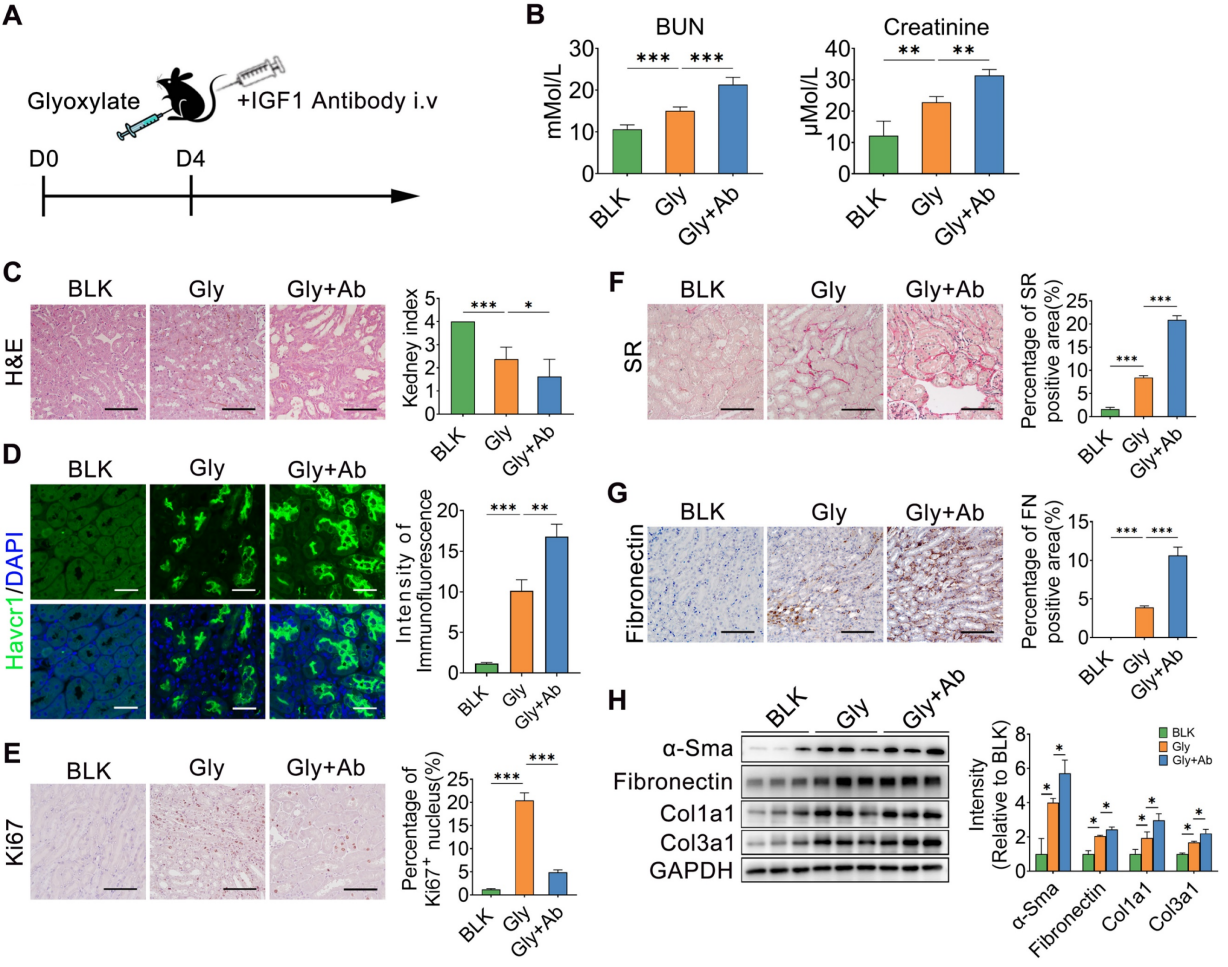
** IGF1 blockade interrupts renal repair in oxalate-induced injury kidney. A** Schematic diagram of the experimental design showing that the IGF1 antibody was administered to mice treated with subsequent glyoxylate for 4 days. **B** The serum BUN and creatinine levels increased after IGF1 antibody treatment in mice pre-administered glyoxylate.** C-D** Images of HE (C) and Havcr1 (D) staining assays revealing tubular injury. **E** Ki67 staining indicating tubular cell proliferation. **F-G** Sirius red (F) and fibronectin (G) staining assays showed exacerbated renal fibrosis after IGF1 antibody treatment. **H** Western blot results showing changes in the abundance of α-SMA, fibronectin, Col1α1 and Col3α1 in several individual experiments. Two-way ANOVA analysis, * *p* < 0.05, ** *p* < 0.01, ***p < 0.001; Scale bar: 50 µm.

The IGF1 antibody was also administered to mice after subsequent glyoxylate insult for 4 days (Figure 8A). As the IGF1 antibody resulted in IGF1 blockade, renal function, as indicated by the serum BUN and creatinine levels, further deteriorated (Figure 8B). The results of HE and Havcr1 immunofluorescence staining revealed more severe renal injury after IGF1 blockade (Figure 8C-D). On the other hand, the Ki67 staining assay showed a decrease in the number of proliferating renal tubule cells after blocking IGF1 (Figure 8E). In addition, the results of the SR and fibronectin staining assays indicated more severe renal fibrosis in the crystallized kidneys after IGF1 antibody treatment (Figure 8F-G). The abundance of fibrotic proteins, including α-Sma, fibronectin, Col1α1, and Col3α1, in mouse kidneys was significantly increased after IGF1 blockade (Figure 8H). Moreover, the number of total macrophages increased in the injured kidney after blocking IGF1 treatment (Figure S13A). However, MRC^+^ macrophages were not the main component in total macrophages (Figure S13B). Thus, IGF1 is required to protect the kidney against injury, allowing the restoration of renal tubular cells to further improve renal function recovery and subsequent fibrosis blockade.

### IGF1 alleviates the NaOx -induced injury in kidney tubule cells via the AKT/Rb signaling pathway

**Figure 9:**
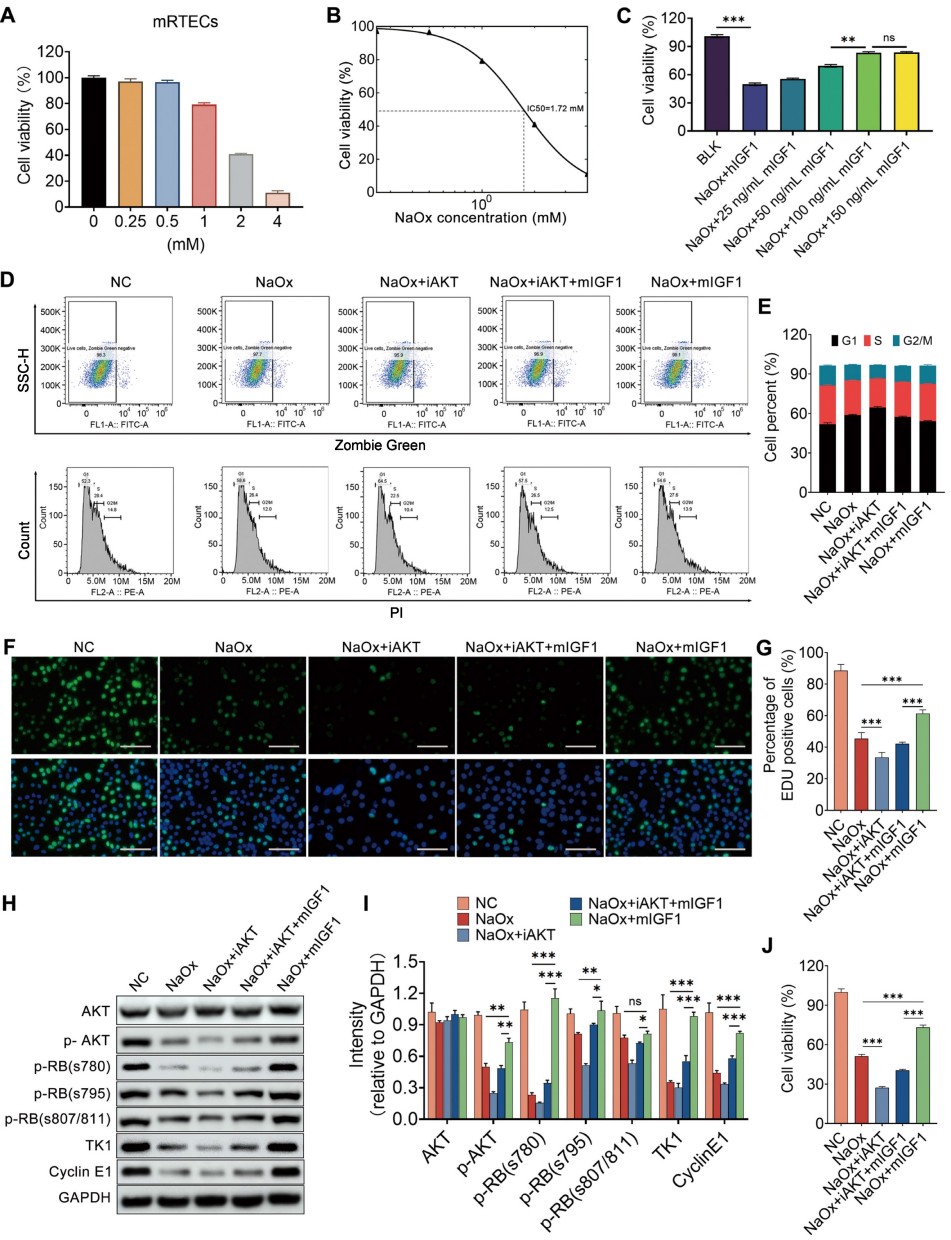
** IGF1 alleviates NaOx-induced injury in kidney tubule cells via the AKT/Rb signaling pathway. A** CCK-8 assay was used to determine the changes in cell viability after oxalate administration at different doses to treat mRTECs. **B** Dose‒response curve indicating that the half-maximal inhibitory concentration (IC50) was approximately 1.72 mM. **C** Effects of different doses of IGF1 on the decrease in mRTECs viability caused by oxalate; two-way ANOVA and Dunnett's multiple comparisons test were used. **p < 0.01, *** *p* < 0.001, ns, not significant. **D-E** Flow cytometry was used to determine changes in the cell cycle distribution of mRTECs after IGF1 administration in response to NaOx-induced injury with or without the inhibition of AKT activity by 10 μm AKT inhibitor VIII (iAKT). The cell cycle status was determined by using propidium iodide staining after distinguishing the dead cells with Zombie dye Green (D). A bar plot showing the change in the percentage of cells in different cell cycle phases (E). **F-G** EdU proliferation assay results showing the growth of mRTECs after NaOx, iAKT and IGF1 administration (F). The cells in green fluorescence are in S-phase. The bar plot shows the percentage changes in cells with respect to green fluorescence (G). Multiple t tests, *** *p* < 0.001. **H-I** Western blot staining results showing that oxalate downregulates the abundance of p-Akt, p-RB (s780, s795, and s807/811), TK1 and Cyclin E1 in mRTECs, while IGF1 administration upregulates them. Multiple t tests, * *p* < 0.05, ** p < 0.01, *** *p* < 0.001. Scale bar: 50 µm. **J** CCK-8 assay showing that iAKT reduced the ability of IGF1 to promote the proliferation of NaOx-induced injured mRTECs.

After finding the impact of IGF1 on injured kidney tubule cells in vivo, both mRTECs and HK2 cells were utilized to verify its effect in vitro. Oxalate induced a sharp decrease in cell viability in a dose-dependent manner (Figure 9A and Figure S14A). The administration of either 1.72 mM NaOx or 0.5 mM NaOx to mRTECs or HK2 cells resulted in an approximately 50% decrease in cell viability for both cells (Figure 9B and Figure S14B), and these doses were chosen for our subsequent experiments. Mouse IGF1 (mIGF1) and human IGF1 (hIGF1) were separately supplemented to kidney tubule cells injured with NaOx, and the results indicated that the vitalities of mRTECs and HK2 cells were restored after individual treatment with either mIGF1 or hIGF1, which were both 100 ng/ml at the optimal doses (Figure 9C and Figure S14C). In addition, the results of flow cytometry analyses indicated that NaOx induced cell cycle arrest at the G1 phase, while IGF1 promoted cell cycle transition to S phase in both mRTECs and HK2 cells (Figure 9D-E and Figure S14D-E). Moreover, the results of the EdU assay showed that IGF1 increased the number of EdU-positive cells, indicating that the cells in the S phase increased (Figure 9F-G and Figure S14F-G). The cell cycle-related proteins Cyclin E1 and TK1 32, 33 were both decreased in oxalate-treated mRTECs and HK-2 cells, but both of these levels were restored after IGF1 treatment, suggesting that IGF1 facilitates the entry of NaOx-injured tubular cells into the S phase (Figure 9H-I and Figure S14H-I).

Because phosphorylation of the retinoblastoma protein (RB) governs the exit from the G1 phase and the transition into the S phase 34, the phosphorylated forms of RB (Ser780, Ser795, and Ser807/811) were detected by western blot, and the results indicated that their expression decreased after NaOx treatment but started to increase after IGF1 treatment (Figure 9H-I and Figure S14H-I). Furthermore, NaOx treatment attenuated the phosphorylation of Akt, while IGF1 treatment maintained Akt phosphorylation at normal levels (Figure 9H-I and Figure S14H-I). In particular, after the activity of AKT was blocked by the 10 µm AKT inhibitor VIII (iAKT), the ability of IGF1 to promote the proliferation of NaOx-induced injured mRTECs was reduced (Figure 9J). However, the number of cells that entered the S phase also decreased (Figure 9D-H). Taken together, these results suggest that IGF1 promotes the G1/S phase transition by activating Akt signaling.

## Discussion

In the present study, we implemented single-cell transcriptomic sequencing technology to generate an atlas of the dynamic changes in kidney cells caused by glyoxylate-induced crystal damage. We observed that a group of tubule cells transitioned into a proliferative state under injury insult, which indicated that a vital process modulates renal self-repair 31. By exploring the factors regulating this process, we identified a group of macrophages that contribute to renal repair in the setting of kidney crystal injury. Consistent with the findings of previous studies, our work illustrated those immune cells, especially macrophages, infiltrate after crystal-induced tubule injury 7, 18, 35. Among them, a transcriptionally distinct macrophage subset increased in number and percentage with crystal damage progression. These cells express the canonical M2 macrophage marker Mrc1 but distinctly secrete the cytokine IGF1, a polypeptide growth factor that plays a key role in regulating cell proliferation, differentiation, metabolism and survival 36, 37. Cellular interaction analysis revealed the significant information flow of IGF1 signal from Mrc1^+^ macrophages to injured and proliferating tubule cells, indicating that these cells play essential roles in tissue repair and kidney restoration in response to crystal insult. A previous study revealed that IGF1 production from alveolar macrophages suppresses endogenous inflammatory signals via the upregulation of phagocytosis by alveolar epithelial cells 36. In our work, IGF1 supplementation enhanced the regeneration capacity of renal tubule cells in glyoxylate-treated mice, ultimately leading to decreased inflammatory and fibrotic responses and decreased macrophage invasion. Conversely, IGF1 blockade exacerbates injury by limiting tubular proliferation and exacerbating inflammation and fibrosis. IGF1/Akt/RB signaling plays a role in enhancing the G1/S transition of the cell cycle and thus promotes tubule cell proliferation. Combined with findings from integrated analysis of other scRNA-seq data on sorted kidney myeloid cells with unilateral IRI insult 16, 38. Based on the markers identified in our data, we observed the conservation of this group of Mrc1^+^ Igf1^+^ macrophages in IRI-challenged kidneys. These data indicate that modulation of Mrc1^+^ Igf1^+^ macrophages could be used as a precise intervention strategy to mitigate crystal nephropathy as well as other kinds of renal injury.

Considering the ontogeny of Mrc1^+^ Igf1^+^ macrophages, their expression characteristics and pseudotime analysis suggest that they are more likely to infiltrate myeloid cells with relatively higher levels of Itgam and proximity to Chil3^+^ macrophages 39, 40. However, their critical origin remains to be determined. Considering the fate of myeloid cells, our results provide insight into the complex microenvironment of injured renal tissues, guiding their differentiation into diverse states and functions. We confirmed the contribution of injured tubule cells to macrophage differentiation into the Mrc1^+^ Igf1^+^ state. For the responsible signals, cellular interaction analysis revealed the intensive involvement of SPP1 and MIF signals. In hepatocellular carcinoma, SPP1 can trigger the polarization of macrophages to M2-phenotype tumor-associated macrophages via the SPP1-CD44 association 41. MIF, a macrophage migration inhibitory factor, is an inflammatory cytokine and an important regulator of the innate immune system 42.

The MIF-CD74 axis is important for macrophages and dendritic cells and plays an immunosuppressive role in tumor progression 43-45. Moreover, several distinct signals directed from tubule cells to Mrc1^+^ macrophages were observed, e.g., the Gas6-Mertk, Fgf1-Fgfr1 and Angptl4-Sdc3 axes. Gas6 is a ligand that binds to the macrophage receptors Tyro3, Axl, and Mertk and skews macrophage polarization toward the M2-like phenotype 46. FGF-1 also skews the polarization of human monocytes toward M2 macrophage, resulting in the elimination of corneal herpetic keratopathy 47. ANGPTL4 blunts the polarization of macrophages toward the proinflammatory phenotype and induces cardiac repair 48. Taken together, multiple cytokines secreted from injured tubule cells contribute to shaping Mrc1^+^ macrophages. However, the specific role of these cytokines in generating the Mrc1^+^ phenotype needs to be further clarified.

Our study identified 15 distinct cell types from steady and crystallized murine kidneys. Similar to prior scRNA-seq studies in which intact kidney samples were used, the majority of the captured cells were of tubular origin, whereas glomerular and mesangial cells were far less represented 14, 49. Additionally, a limited number of immune cells, especially myeloid cellsa mere 1812 cells, were captured. Despite the supplementation of other scRNA-seq data from sorted kidney myeloid cells, our analysis still omits information regarding the dynamic origins, states and functions of myeloid cells in the renal response to crystal injury. To address these issues, future studies are needed to perform diverse cell capture and illustrate more details, including spatial information, on disease progression.

This study provides a cellular atlas of glyoxylate-induced crystal nephropathy and reveals important crosstalk between macrophages and the epithelium that dominates kidney regeneration after injury. Our results help to elucidate tubular and immune cell responses to crystal injury and may lead to the identification of potential cellular and molecular targets for the treatment of crystal nephropathy.

## Supplementary Material

Supplementary methods and figures.

Supplementary table 1.

Supplementary table 2.
