# Supplementary material for: Mrc1+ macrophage-derived IGF1 mitigates crystal nephropathy by promoting renal tubule cell proliferation via the AKT/Rb signaling pathway
Source: Theranostics. 2024 Feb 17;14(4):1764–80. doi: 10.7150/thno.89174 (PMC10879870; doi:10.7150/thno.89174)
Supplement: Supplementary file 1 — Supplementary methods and figures. [file thnov14p1764s1.pdf]

Supplementary Information

Mrc1$^+$ macrophage-derived IGF1 mitigates crystal nephropathy by promoting renal tubule cell proliferation *via* the AKT/Rb signaling pathway

Linxi Huang, Wei Chen, Zhuojing Tan, Yunxiao Huang, Xinji Gu, Lantian Liu, Hongxia Zhang, Yihan Shi, Jiarong Ding, Chengjian Zheng, Zhiyong Guo, and Bing Yu

List of Supplementary Material

1) Supplementary Methods

2) Supplementary Figures

Figure S1, related to Figure 1

Figure S2, related to Figure 1

Figure S3, related to Figure 2

Figure S4, related to Figure 2

Figure S5, related to Figure 3

Figure S6, related to Figure 4

Figure S7, related to Figure 5

Figure S8, related to Figure 5

Figure S9, related to Figure 5

Figure S10, related to Figure 6

Figure S11, related to Figure 6

Figure S12, related to Figure 6

Figure S13, related to Figure 8

Figure S14, related to Figure 9

3) Supplementary Tables

Table S1: DEGs of 15 clusters generated by Findallmarkers.

Table S2: DEGs of 6 sub-clusters of myeloid cells generated by Findallmarkers.

1) Supplementary Methods

Single-cell RNA sequencing and analysis

Mice kidneys were harvested and dissected in sterile culture dish with 10 ml 1X Dulbecco's Phosphate-Buffered Saline (DPBS, Thermo Fisher, 14190144) on ice to remove the tissue storage solution, then minced on ice. Tissues were digested with 0.25% Trypsin (Thermo Fisher, 25200-072) and 10 μg/ml DNase I (Sigma, 11284932001) dissolved in PBS with 5% Fetal Bovine Serum (FBS, Thermo Fisher, SV30087.02). Tissues were dissociated at 37℃ with a shaking speed of 50 rpm for about 40 minutes. Cells were repeatedly collected at interval of 20 minutes to increase cell yield and viability. Cell suspensions were filtered using 40 μm nylon cell strainer and red blood cells were removed by 1X Red Blood Cell Lysis Solution (Thermo Fisher, 00-4333-57). Dissociated cells were washed with 1 X DPBS containing 2% FBS. Cells were stained with 0.4% Trypan blue (Thermo Fisher, 14190144) to check the viability on Countess® II Automated Cell Counter (Thermo Fisher) [1].

10X library preparation and sequencing

Beads with unique molecular identifier (UMI) and cell barcodes were loaded close to saturation, so that each cell was paired with a bead in a Gel Beads-in-emulsion (GEM). After exposure to cell lysis buffer, polyadenylated RNA molecules hybridized to the beads. Beads were retrieved into a single tube for reverse transcription. On cDNA synthesis, each cDNA molecule was tagged on the 5' end (ie, the 3' end of a messenger RNA transcript) with UMI and cell label indicating its cell of origin. Briefly, 10X beads that were then subject to second-strand cDNA synthesis, adaptor ligation, and universal amplification. Sequencing libraries were prepared using randomly interrupted whole-transcriptome amplification products to enrich the 3' end of the transcripts linked with the cell barcode and UMI. All the remaining procedures including the library construction were performed according to the standard manufacture's protocol (CG000206 Rev D). Sequencing libraries were quantified using a High Sensitivity DNA Chip (Agilent) on a Bioanalyzer 2100 and the Qubit High Sensitivity DNA Assay (Thermo Fisher). The libraries were sequenced on NovaSeq6000 (Illumina) using 2X150 chemistry.

Mapping

Reads were processed using the Cell Ranger 2.1.0 pipeline with default and recommended parameters. FASTQs generated from Illumina sequencing output were aligned to the mouse

genome, version GRCm38, using the STAR algorithm [2]. Gene-Barcode matrices were generated for each sample by counting UMIs and filtering non-cell associated barcodes. Finally, we generate a gene-barcode matrix containing the barcoded cells (D0, 16426; D1, 12730; D4, 16427; D7, 15087) and gene expression counts.

Pre-processing and quality control

The raw transcript counts of gene-cell matrices of 4 samples were imported to R software and package Seurat (version 3.1.0) processed subsequent analysis [3]. Genes expressed in less than 3 cells were removed. Low-quality cells with less than 300 expressed genes were excluded. Cells expressing a high number of genes (> 3500 - 4300) were considered as putative doublets or multiples and were removed. Cells with a high percentage of transcripts mapping to mitochondrial genes (> 32.5%) were regarded as dead or lysed and were removed. Datasets of four samples were pre-processed and filtered individually. Without noticing a significant batch effect across 4 samples, we directly merged the 4 datasets as 1 matrix for subsequent analysis using “merge” function in Seurat.

Normalization, dimensionality reduction, clustering and nomenclature

The UMI counts matrices were then log normalized and scaled with Seurat’s NormalizeData and ScaleData functions. The influence of nCount_RNA and the percent of mitochondrial genes were regressed within the ScaleData function. 2000 genes with the highest variance were used to perform linear dimensional reduction (principal component analysis), and the number of principal components used in downstream analyses was chosen considering Seurat’s Elbowplot. We screened all the dims around the Elbowpoint to determine the best dim to be chosen. The final dim was set at 25. Seurat’s unsupervised graph-based clustering was performed on the projected PC space. Findneighbors function was performed to cluster cells and different resolutions were screened to determine the best one. The final resolution was set at 0.2. t-distributed stochastic neighbor embedding (tSNE) was performed for data visualization of different cell clusters in two-dimensional space. tSNE map displayed the distribution of 4 datasets and indicated no significant batch effect. Findallmarkers function configured with Wilcoxonsigned-rank statistical test was applied to calculate significantly differentially expressed genes (DEGs) of each cluster. Parameters for figuring out DEGs were set as min.pct: 0.25; logFC threshold: 0.25; adjusted p value < 0.05. Known marker genes identified from published single-cell sequencing data were referred to

annotate our cell clusters [4]. Heatmap displays the average expression of DEGs in each cluster.

After the identification of myeloid cells, the process was repeated for clustering and annotating sub-clusters of myeloid cells. Top 10 DEGs ranked by average expression logFC of each cluster are shown in heatmap.

Trajectory analysis

R package Monocle 2 was performed on kidney epithelial cells, including proximal tubule cells, collecting duct cells and loop of henle cells, and sub-clusters of myeloid cells. Ordering genes were assigned according to marker genes originated from FindAllMarkers function. Data were processed by DDRTree algorithm for reducing the dimension and pseudotime was calculated for each cell. Trajectory chart displays the distribution of different cell types.

Proximal tubule-myeloid cell communication using CellChat

R package CellChat was utilized to infer potential intercellular communication between 2 dynamic proximal tubule cells and myeloid sub-types [5]. The settings were all default in our data processing. We first identified significant ligand-receptor pairs mediating signals. Then CellChat figured out significant information flows. CellChat also revealed both incoming and outgoing communication patterns of selected cells. To compare cellular interactions across samples, the analysis was then conducted separately on data D1 and D7. Then the inferred cellular interactions were merged to visualize the differences.

Functional Enrichment Analysis

DEGs of proliferation PT cells versus PT cells were obtained by Findmarkers function in Seurat. Heatmap displays the top 30 up-regulated or down-regulated DEGs ranked by average logFC. Upregulated DEGs of proliferating PT cells were used for functional enrichment via online server KOBAS [6]. DEGs of myeloid sub-types were obtained by Findallmarkers and only positive results were kept. Metascape and R package clusterProfiler (version 4.0.5) were used for functional enrichment to illustrate the functional differences of each category.

Online data analysis

The first 15 integrated principal components were chosen as input for Uniform Manifold Approximation and Projection (UMAP) reduction and resolution 0.3 were selected for clustering cells. DEGs were identified by FindAllMarkers function with default parameters on the normalized SCT data. Top 30 DEGs identified in Mrc1+ macrophages in our sequencing data were used as

gene set to score kidney mononuclear phagocytes in this dataset. AddModuleScore function in Seruat package calculated scores of every mononuclear phagocyte. Visualization of gene expression or score by scatter plots and violin plots were implemented through function FeaturePlot and VlnPlot in the Seurat package.

Immunohistochemistry and Immunofluorescence

Tissue slides received Tris-EDTA (pH 9.0) antigen retrieval at 121°C for 2 minutes, then were blocked with 3% H_2O_2 for 10 min at room temperature to block the endogenous peroxidase activity. The non-specific binding site was blocked with 1% BSA for 30 min at room temperature. The sections were incubated with primary antibodies at 4 °C overnight. For immunohistochemistry (IHC) staining, the sections were incubated with HRP-conjugated secondary antibodies at 37°C for 30 min, and visualized with DAB Substrate (34,002, Thermo Fisher). For immunofluorescence (IF) staining, the sections were incubated with fluorescein-conjugated secondary antibodies at 37°C for 30 min, then the nuclei were counter stained with DAPI. The primary antibodies include: IGF1 (ab223567, diluted 1:200) from Abcam, MRC1(#24595, diluted 1:200), F4/80 (#70076, diluted 1:200) from Cell Signaling Technology, and Ki67 (27309-1-AP, diluted 1:1000) from Proteintech. The secondary antibodies are listed as follows: Peroxidase AffiniPure goat anti-rabbit IgG (H+L) (111-035-003, diluted 1:200), Peroxidase AffiniPure goat anti-mouse IgG (H+L) (115-035-003, diluted 1:200), Cy™3 AffiniPure goat anti-rabbit IgG (H+L) (111-165-003, diluted 1:200) and Fluorescein AffiniPure goat anti-rabbit IgG (H+L) (111-165-003, diluted 1:200) from Jackson ImmunoResearch.

Western Blot

Kidney tissues and cells were lysed in RIPA buffer (89,901, ThermoFisher) for Western blotting. Protein concentration was quantified by BCA kit (23,225, ThermoFisher). Twenty micrograms of total protein per lane were loaded and separated by SDS-PAGE electrophoresis, then the proteins were transferred to PVDF membranes. The membranes were blocked with 5% skimmed milk powder for 30 min, and incubated with a primary antibody at 4°C overnight. The membranes were washed with TBST for three times, and incubated with an HRP-conjugated secondary antibody at 37°C for 1 h. The immune complexes were visualized by using Pierce™ ECL Western Blotting Substrate (32,132, ThermoFisher). The primary antibodies are listed as follows: IGF1 (#73034,

diluted 1:1000), α-SMA (#19245, diluted 1:1000) from Cell Signaling Technology, beta actin (66,009-1-Ig, diluted 1:5,000), and Alpha Tubulin (66,031-1-Ig, diluted 1:5,000) from Proteintech.

Real-Time PCR

Total RNA was purified with the RNAiso Plus reagent (9,108, Takara). Then, cDNA was synthesized by the MMLV Reverse Transcriptase (M530A, Promega) according to the manufacturer's protocols. A real-time PCR analysis was performed on the LightCycler® 96 System (Roche) with ChamQ SYBR qPCR Master Mix (Q321-02, Vazyme) according to manufacturer's suggesting protocol. The primers were summarized in the Table 1. All samples were examined thrice. The fold changes of each target gene were calculated using the $2^{-\triangle\triangle Ct}$ method relative to GAPDH.

Table 1 The primers for Real-Time PCR

Primer name	Sequences
Gapdh-forward	AGGTCGGTGTGAACGGATTTG
Gapdh-reverse	TGTAGACCATGTAGTTGAGGTCA
Igf1-forward	CTGGACCAGAGACCCTTTGC
Igf1-reverse	GGACGGGGACTTCTGAGTCTT
Mrc1-forward	CTCTGTTCAGCTATTGGACGC
Mrc1-reverse	CGGAATTTCTGGGATTCAGCTTC
Arg1-forward	CTCCAAGCCAAAGTCCTTAGAG
Arg1-reverse	AGGAGCTGTCATTAGGGACATC
Il10-forward	GCTCTTACTGACTGGCATGAG
Il10-reverse	CGCAGCTCTAGGAGCATGTG
Havcr1-forward	ACATATCGTGGAATCACAACGAC
Havcr1-reverse	ACTGCTCTTCTGATAGGTGACA
Acta2-forward	GTCCCAGACATCAGGGAGTAA
Acta2-reverse	TCGGATACTTCAGCGTCAGGA
Col3a-forward	ACGTAGATGAATTGGGATGCAG
Col3a-reverse	GGGTTGGGGCAGTCTAGTG
Col1a1-forward	GCTCCTCTTAGGGGCCACT
Col1a1-reverse	CCACGTCTCACCATTGGGG

Reference

1. Macosko EZ, Basu A, Satija R, Nemesh J, Shekhar K, Goldman M, et al. Highly Parallel Genome-wide Expression Profiling of Individual Cells Using Nanoliter Droplets. Cell. 2015, 161(5):1202-14. doi:10.1016/j.cell.2015.05.002
2. Le DT, Durham JN, Smith KN, Wang H, Bartlett BR, Aulakh LK, et al. Mismatch repair deficiency predicts response of solid tumors to PD-1 blockade. Science. 2017, 357(6349):409-13. doi:10.1126/science.aan6733
3. Butler A, Hoffman P, Smibert P, Papalexi E, Satija R. Integrating single-cell transcriptomic data across different conditions, technologies, and species. Nat Biotechnol. 2018, 36(5):411-20. doi:10.1038/nbt.4096
4. Park J, Shrestha R, Qiu C, Kondo A, Huang S, Werth M, et al. Single-cell transcriptomics of the mouse kidney reveals potential cellular targets of kidney disease. Science. 2018, 360(6390):758-63. doi:10.1126/science.aar2131
5. Jin S, Guerrero-Juarez CF, Zhang L, Chang I, Ramos R, Kuan CH, et al. Inference and analysis of cell-cell communication using CellChat. Nat Commun. 2021, 12(1):1088. doi:10.1038/s41467-021-21246-9
6. Xie C, Mao X, Huang J, Ding Y, Wu J, Dong S, et al. KOBAS 2.0: a web server for annotation and identification of enriched pathways and diseases. Nucleic Acids Res. 2011, 39(Web Server issue):W316-22. doi:10.1093/nar/gkr483

2) Supplementary Figures

Figure S1, related to Figure 1

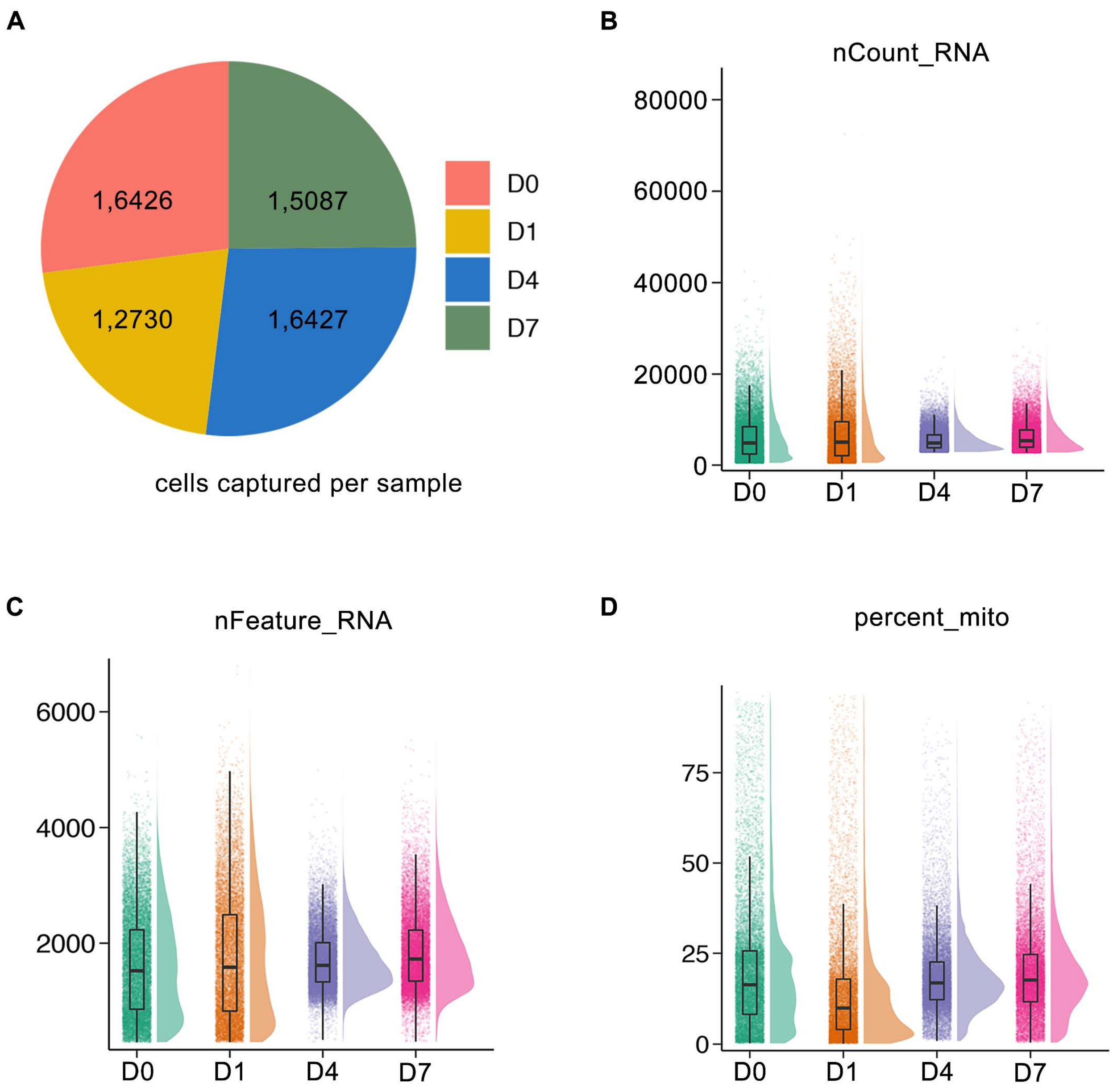

Figure S1. Quality control of 4 sequencing samples. **(A)** Total cells captured in each sequencing sample. **(B-D)** Combined boxplots and vlnplots depict the number of counts (B), genes (C) and percentage of mitochondrial genes (D) in each sequencing sample.

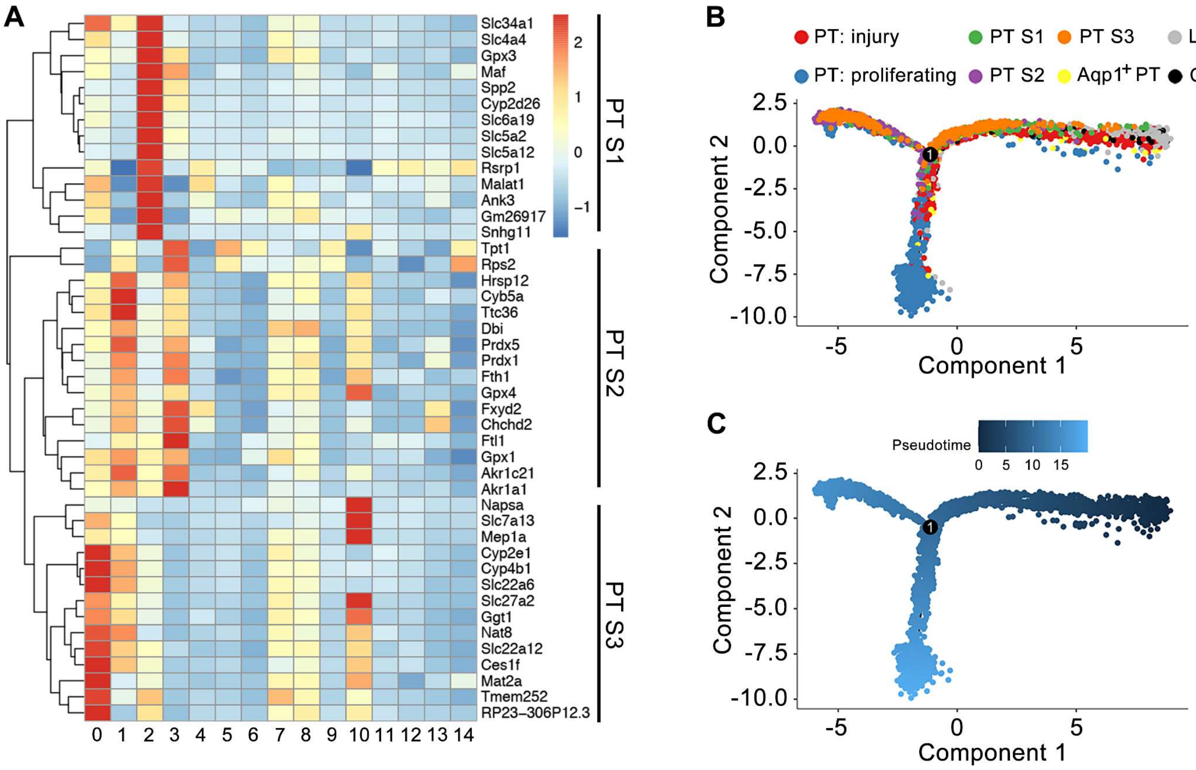

Figure S2, related to Figure 1

Figure S2. Identification and monitoring change of discrete kidney epithelial cell types. (A) Heatmap of single cell-type specific average expression of marker genes, reported in GSE107585, in different segments of proximal tubule cells. Mean expression values of genes were calculated in each cluster (defined in Figure 1). The colour scheme is based on z-score distribution. **(B-C)** Trajectory analysis of kidney epithelial cell populations on glyoxylate treated day 0, 1, 4 and 7. Cells color-labelled by cell type (B) and pseudotime (C) indicating their transition to proliferative state. Number 1 represents the significant branch points of differentiation.

Figure S3, related to Figure 2

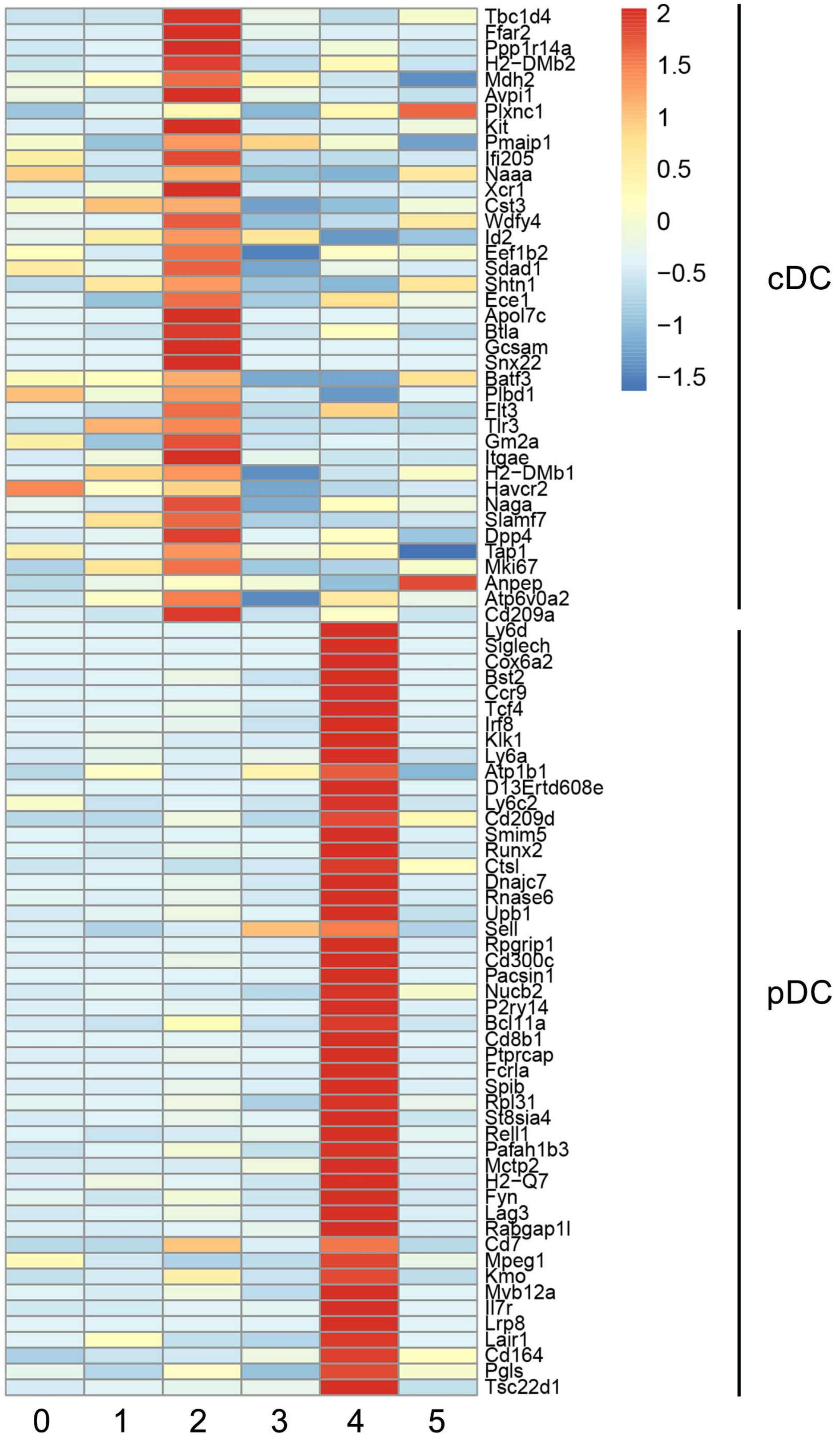

Figure S3. Heatmap shows expression patterns of cDC and pDC marker genes across myeloid sub-clusters.

Figure S4, related to Figure 2

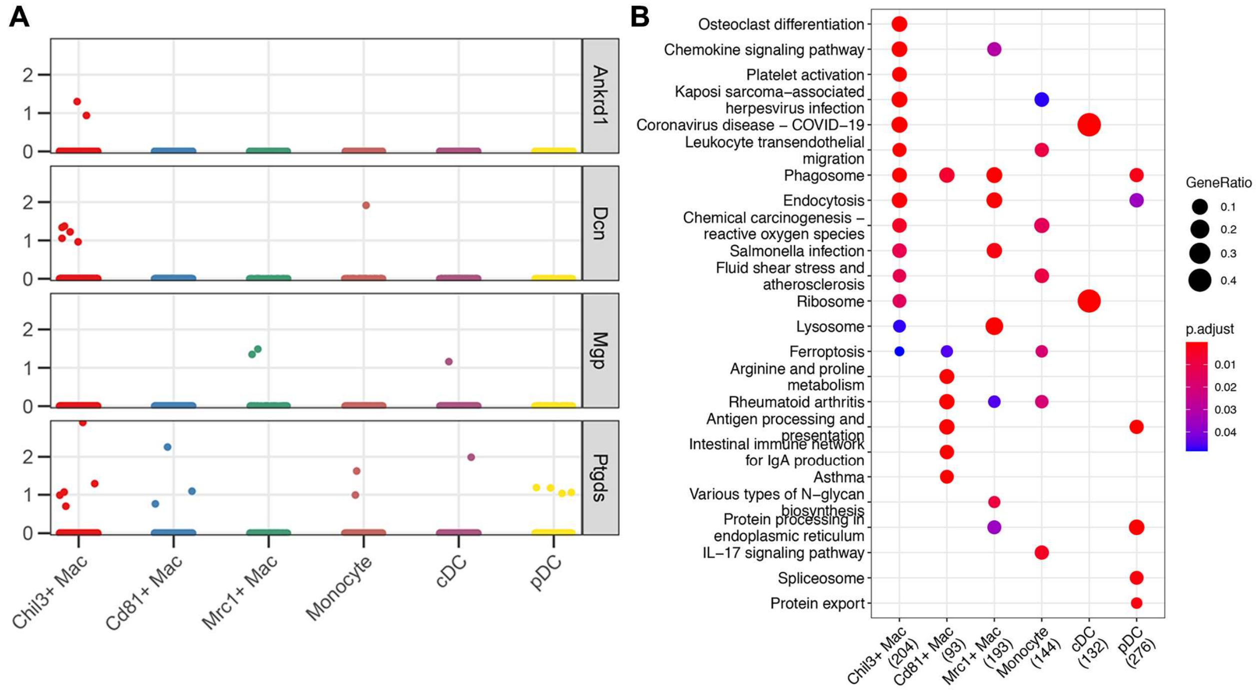

Figure S4. Identification of discrete myeloid subtypes and analyses on their functions. (A) Jitter plot indicating the low expression level of fibrosis related genes in myeloid sub-clusters. (**B)** Dotplot showing the comparison of KEGG enrichment of different myeloid sub-clusters.

Figure S5, related to Figure 3

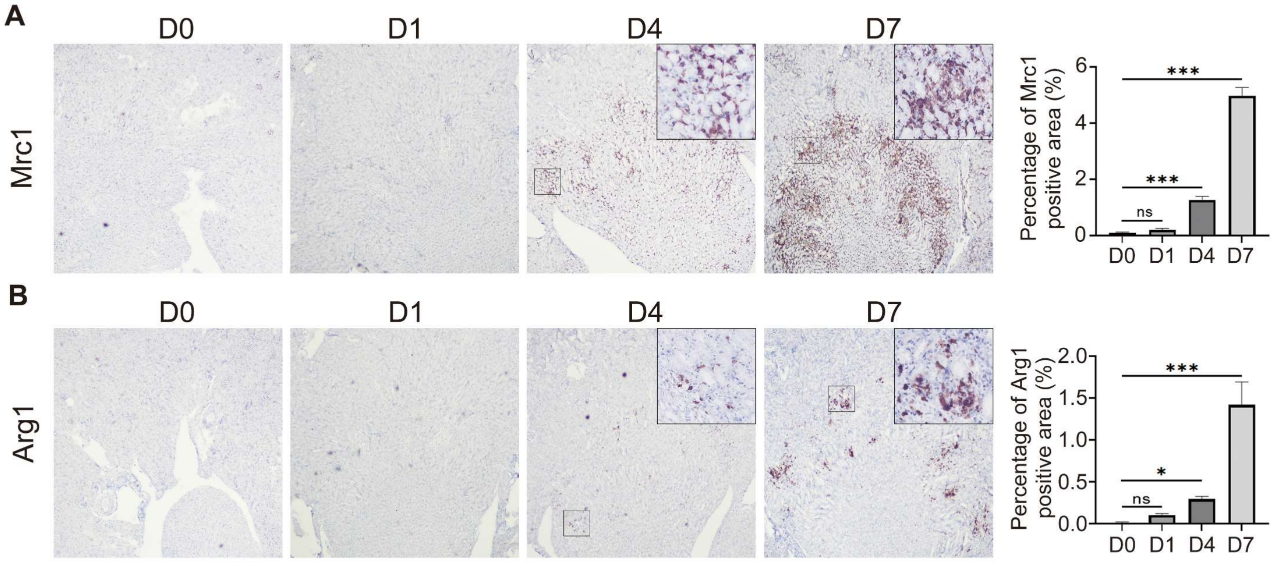

Figure S5. Mrc1 positive or Arg1 positive macrophages increased with crystal nephropathy progression. **(A)** Immunohistochemistry staining of Mrc1 on kidneys collected on D0, D1, D4 and D7 after glyoxylate treatment. Bar plots depicting the change of average optical density of Mrc1 staining. **(B)** Immunohistochemistry staining of Arg1 on kidneys collected on D0, D1, D4 and D7 after glyoxylate treatment. Bar plots depicting the change of average optical density of Arg1 staining. One-way ANOVA analysis, *, $p < 0.05$, ***, $p < 0.001$; ns, not significant.

Figure S6, related to Figure 4

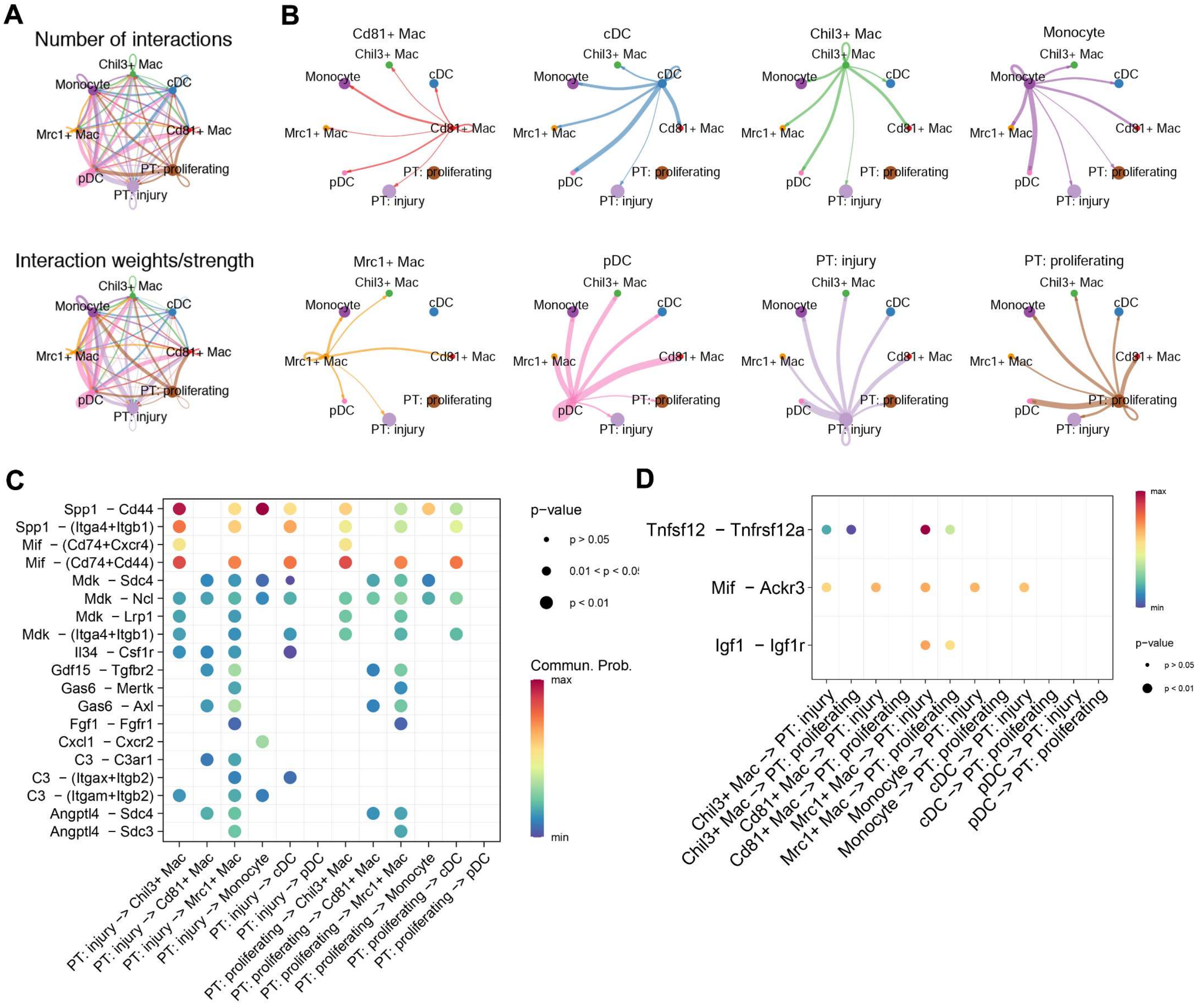

Figure S6. Intercellular communications between 6 types of myeloid cells and 2 types of dynamic tubule cells. **(A)** Circle plots displaying putative ligand-receptor interactions between 6 myeloid cell subtypes and 2 dynamic tubule cell types: injury and proliferating PTs. **(B)** Circle plots displaying the brand link pairs of interacting cell types and corresponding numbers of events. **(C)** Dotplot showing all the significant ligand-receptor pairs that contribute to the signaling sending from injury and proliferating PTs to 6 myeloid states. **(D)** Dotplot showing all the significant ligand-receptor pairs that contribute to the signaling sending from 6 myeloid states to injury and proliferating PTs. The dot color and size represent the calculated communication probability and *p*-values. Empty space means the communication probability is zero. *p*-values are computed from one-sided permutation test.

Figure S7, related to Figure 5

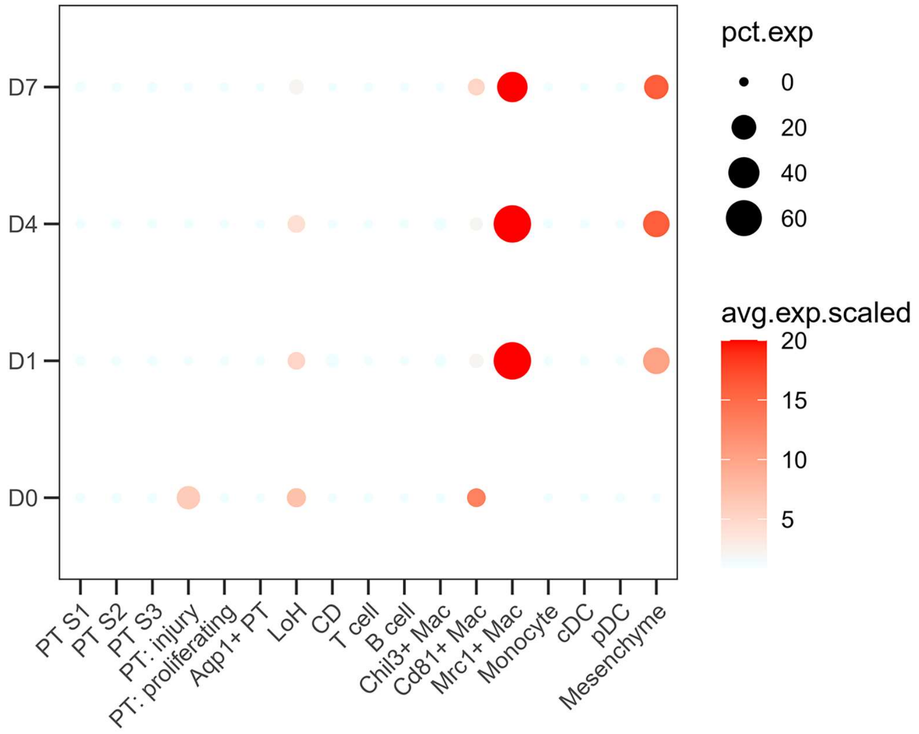

Figure S7. Dotplot shows the Igf1 expression pattern across all cell types from D0 to D7.

Figure S8, related to Figure 5

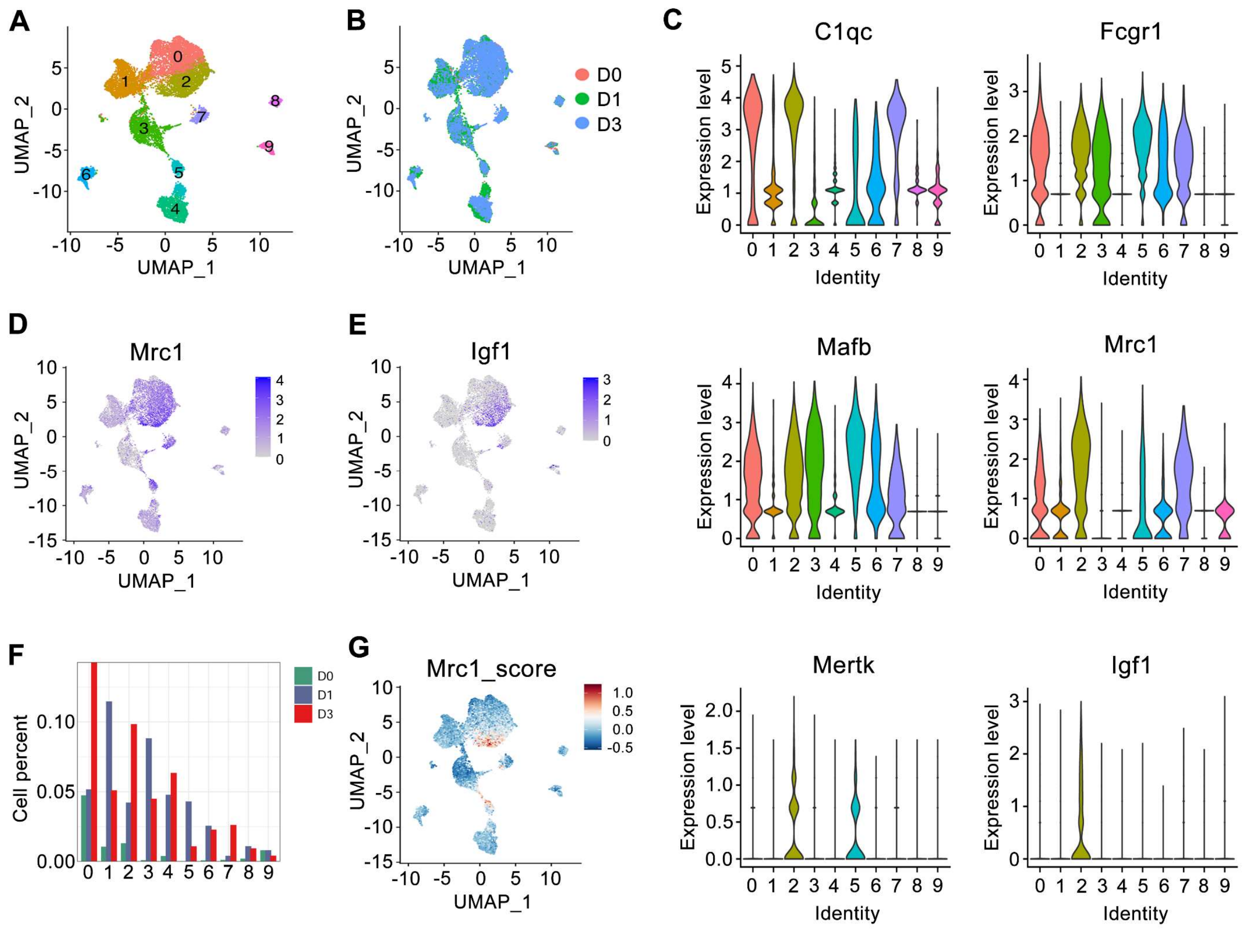

Figure S8. Identification of Mrc1+Igf1+ macrophages in IRI induced renal injury. **(A)** The uniform manifold approximation and projection (UMAP) map of kidney macrophages acquired from GSE174324 indicates 10 cell clusters. **(B)** UMAP plot of kidney macrophages in GSE174324 colored according to experiments exhibits weak batch effect. **(C)** VInplots depict the expression level of representative genes across different macrophage clusters. Y-axis is log scale normalized read count. **(D-E**) Feature plots depict the expression distribution and level of Mrc1 (D) and Igf1 (E) in kidney macrophages. Color scale is log_{10} expression levels of specific genes. **(F)** The percentage change of different clusters of kidney macrophages across experiments. **(G)** UMAP plot depicts the scores of kidney macrophages. Top 30 DEGs of Mrc1+ macrophage generated in our sequencing data were utilized to score cells.

Figure S9, related to Figure 5

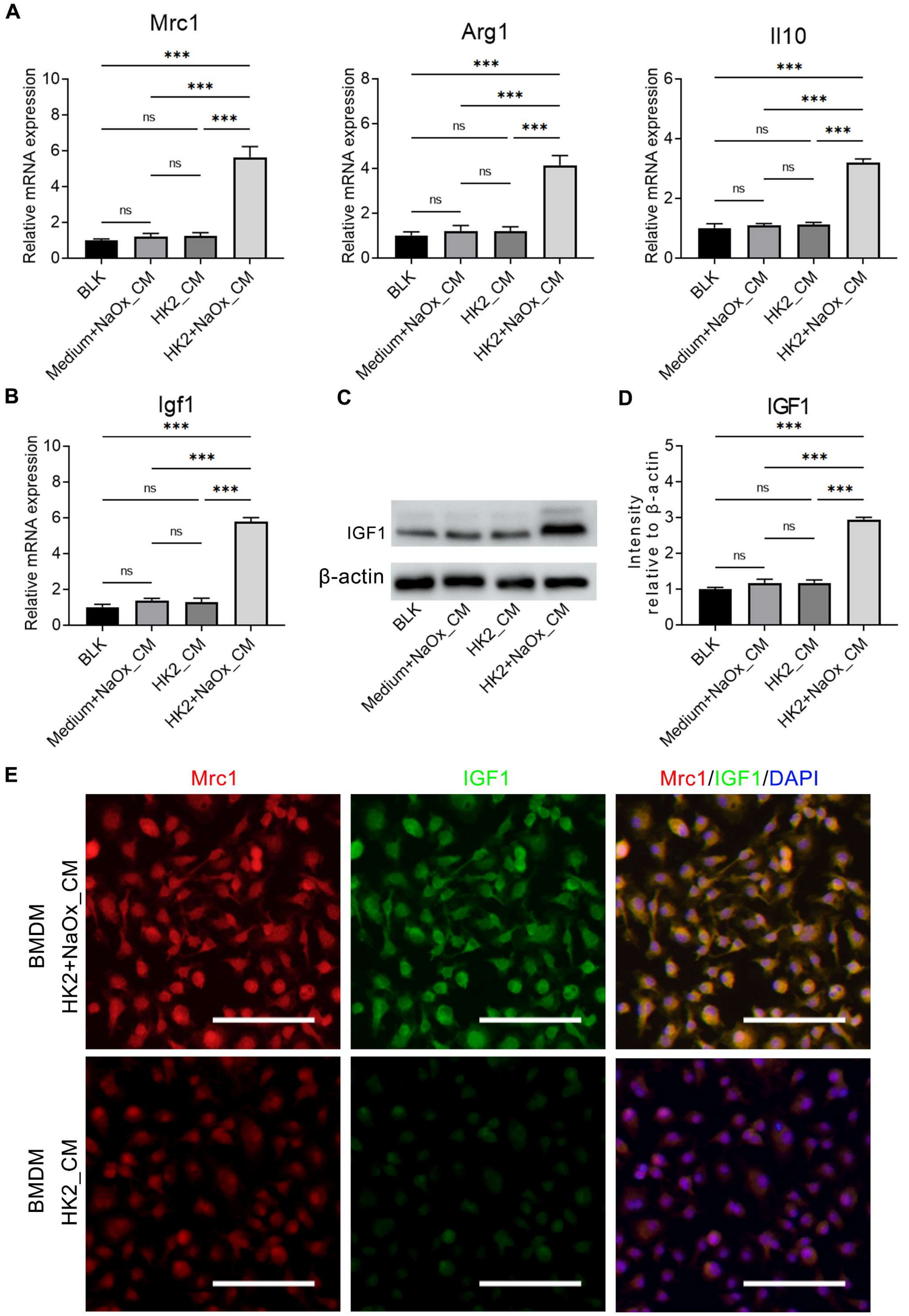

Figure S9. The conditional medium prepared form oxalate-challenged human HK2 cells promotes BMDM to upregulate IGF1 expression along with to enhance the Mrc1⁺ phenotype.

(A) Quantitative PCR shows fold changes for Mrc1, Arg1 and Il10 expressions in the BMDMs treated by the conditioned medium with NaOx treatments. The control medium was prepared from medium of HK2 cells without NaOx treatment or medium treated with NaOx without HK2 cells. **(B)** Quantitative PCR shows Igf1 expression change in BMDMs. (**C-D)** Western blot assay shows IGF1 protein change in BMDMs. **(E)** Immunofluorescence staining assay shows the protein changes for MRC1 and IGF1 in BMDMs treated with the conditioned medium prepared from culture of HK-2 cells with or without NaOx treatments. Multiple t tests, ***, $p < 0.001$; ns, not significant; Scale bar: 50 μm.

Figure S10, related to Figure 6

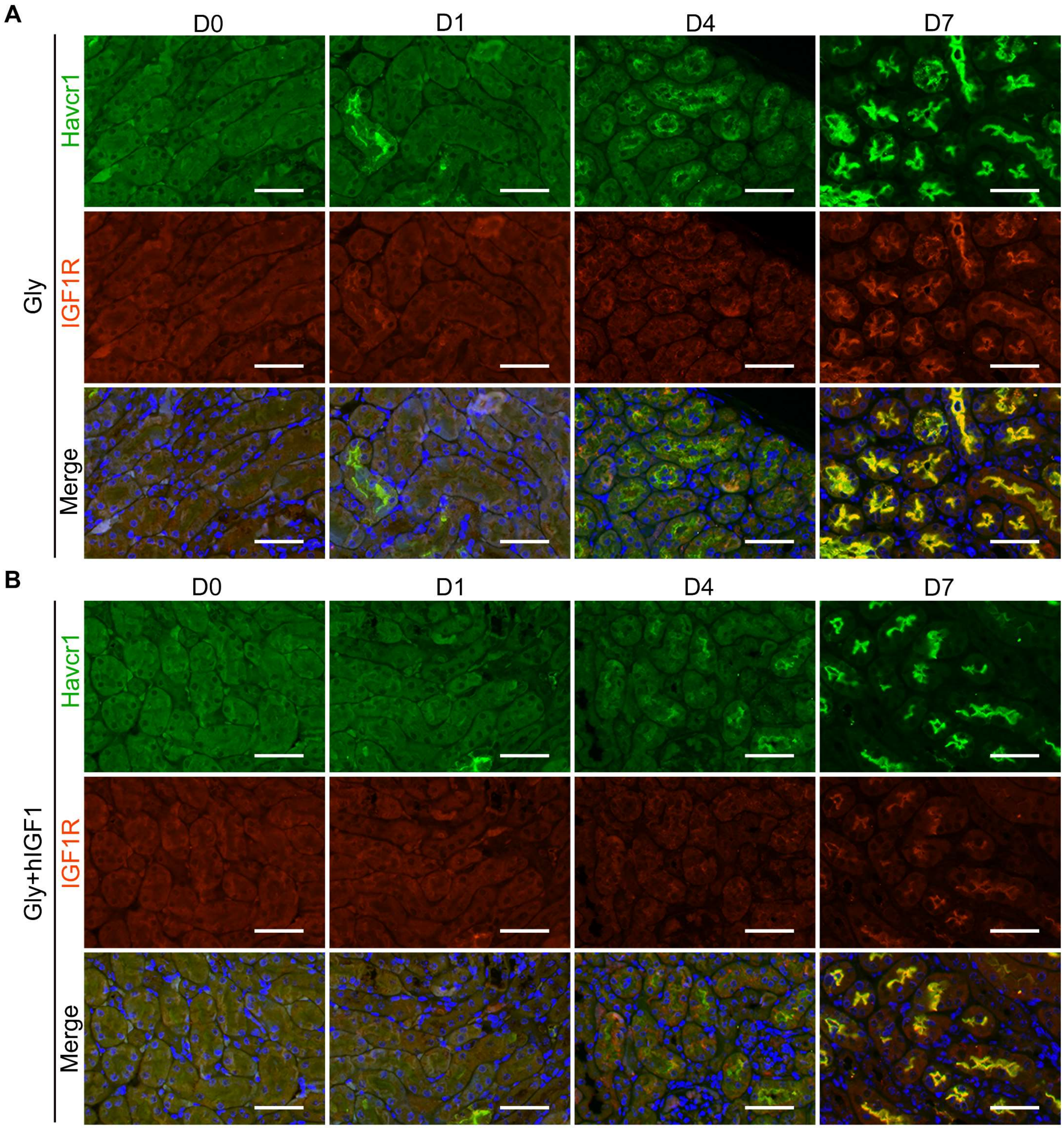

Figure S10. Gly-induced injury tubule cells up-regulate the expression of IGF1 receptor (IGF1R). The results of Co-immunofluorescence staining of Havcr1 and IGF1R showed that the expression of Havcr1, a marker of renal tubular epithelial cell injury, increases as the injury progresses. At the same time, Havcr1-positive injured tubular cells begin to express IGF1R **(A)**, while the administration of hIGF1 significantly reduced the expression of Havcr1 **(B)**. Scale bar: 100 μm.

Figure S11, related to Figure 6

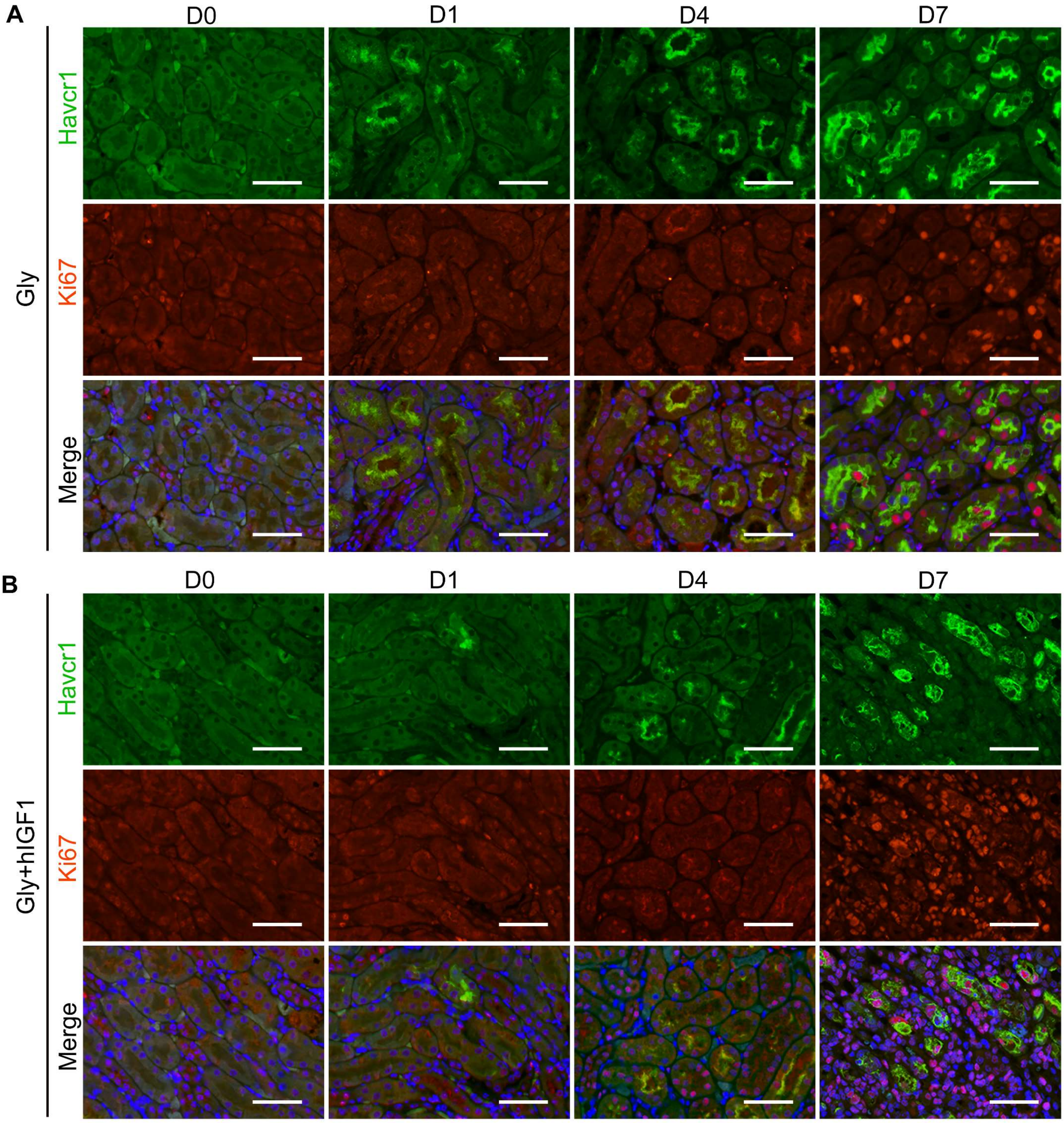

Figure S11. The administration of hIGF1 promotes the proliferation of Gly-induced injury tubule cells. The results of Co-immunofluorescence staining of Havcr1 and Ki67 in the Gly-induced injury group **(A)** and hIGF1 treated Gly-induced injury group **(B)**, indicating that the administration of hIGF1 increased the numbers of Ki67 positive cells in Gly-induced crystal mouse models. Scale bar: 100μm.

Figure S12, related to Figure 6

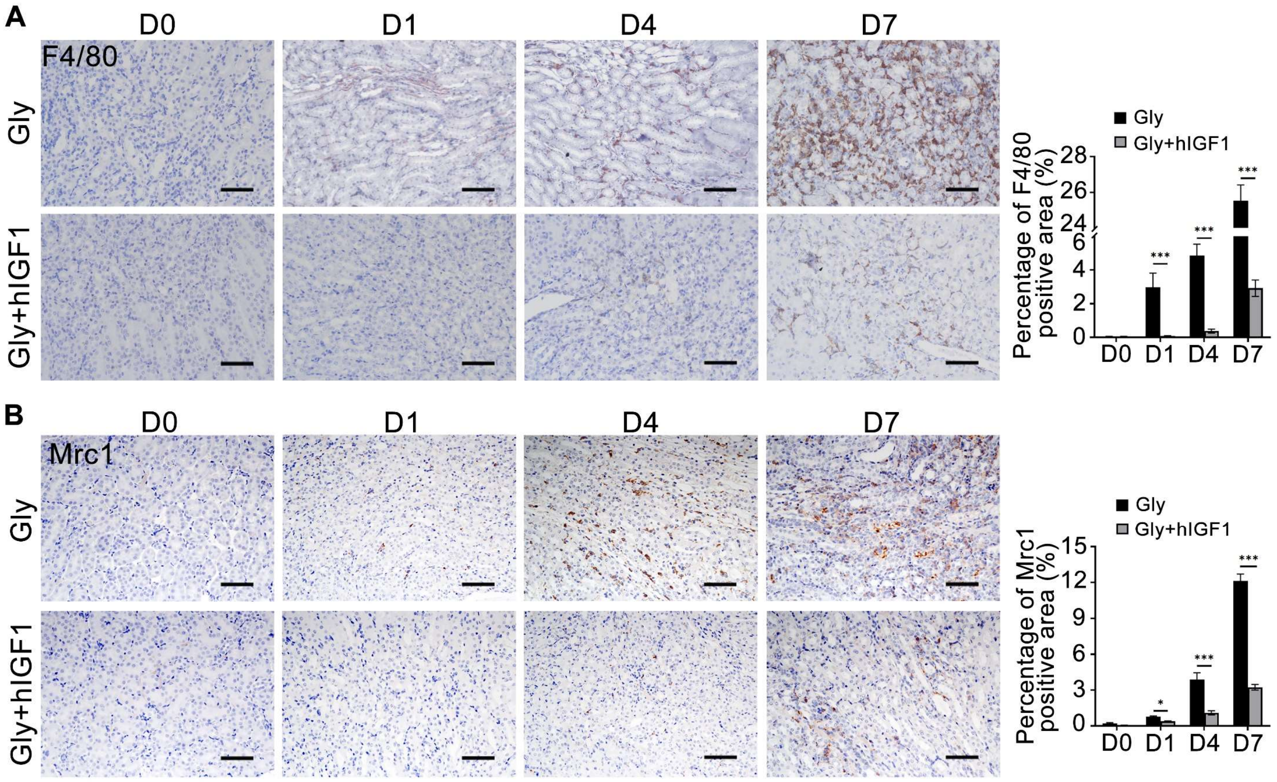

Figure S12. The number of F4/80 and Mrc1 positive macrophages is decreased by IGF1 administration during Gly-induced crystal nephropathy. **(A-B)** Immunohistochemistry staining of F4/80 (A) and Mrc1 (B) on kidneys collected on D0, D1, D4, and D7 after glyoxylate and IGF1 treatment. Bar plots depict the change of average percentage of F4/80 and Mrc1 positive area and the results suggested that IGF1 reduced the number of macrophages in Gly-induced crystal nephropathy. Two-way ANOVA analysis, *$p < 0.05$, *** $p < 0.001$; Scale bar: 100μm.

Figure S13, related to Figure 8

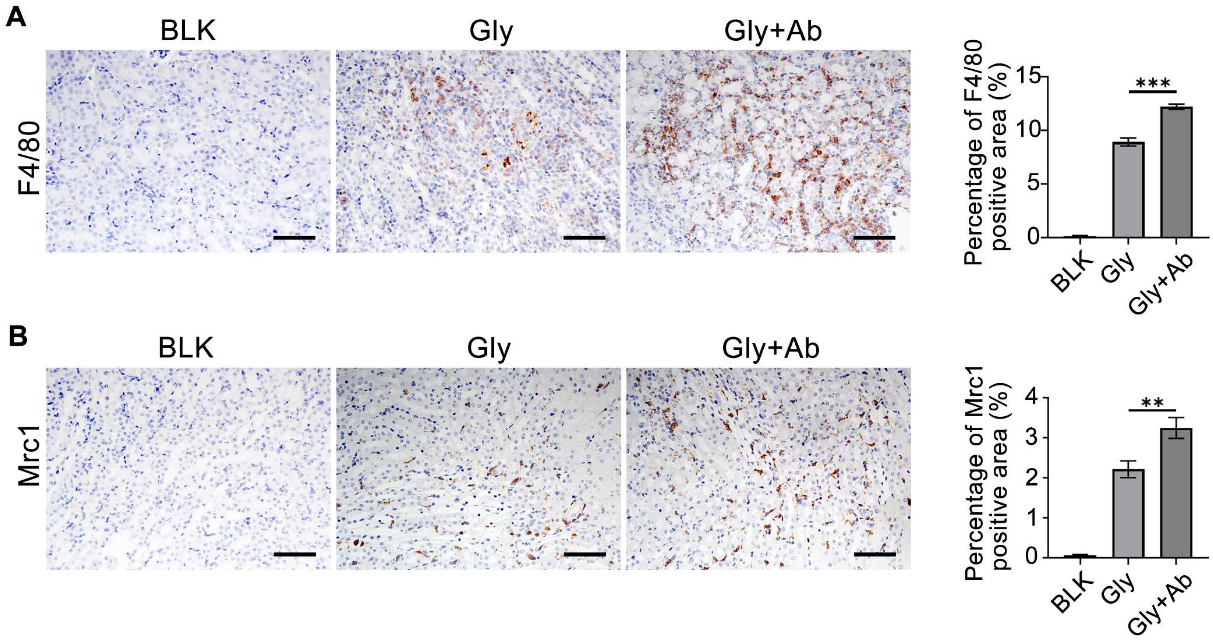

Figure S13. The number of F4/80 and Mrc1positive macrophages is increased by the blockage of IGF1 with antibody during Gly-induced crystal nephropathy. **(A-B)** Immunohistochemistry staining of F4/80 (A) and Mrc1 (B) on kidneys samples from the control group (BLK), Gly-induced crystal group (Gly), and the blockage of IGF1 with antibody in Gly-induced crystal nephropathy group (Gly+Ab). Bar plots depict the change of average percentage of F4/80 and Mrc1 positive area and the results suggested that blockage of IGF1 increased the number of macrophages in Gly-induced crystal nephropathy. Two-way ANOVA analysis, ** $p < 0.01$, *** $p < 0.001$; Scale bar: 100μm.

Figure S14, related to Figure 9

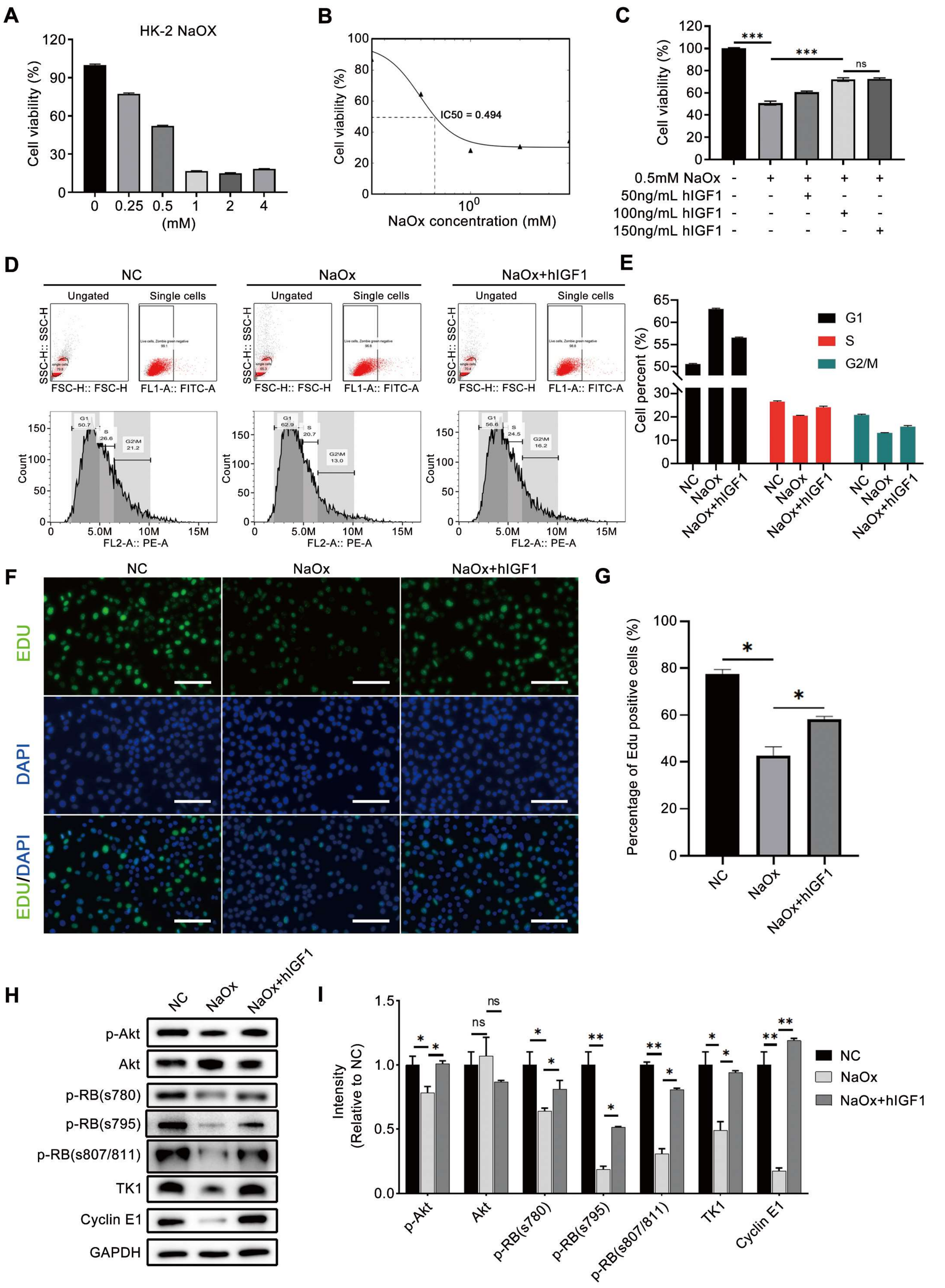

Figure S14. IGF1 suppresses oxalate-induced injury to human HK2 cells. **(A)** CCK-8 assay

was performed to determine the cell viability change after oxalate administration at different doses to HK-2 cells. **(B)** The concentration-response curve indicates the half inhibitory concentration (IC50) at about 0.5mM. **(C)** Effects of different doses of IGF1 on HK-2 cell viability decline caused by oxalate. **(D-E)** Flow cytometry depicts the HK-2 cell cycle distribution change after IGF1 administration to oxalate-induced injury. Cell cycle status was determined using propidium iodide staining after distinguishing the dead cells with Zombie dye Green (D). The bar plot shows the cell percentage change of cells in different cell cycle phases (E). **(F-G)** EDU proliferation assay analysis of the growth of HK-2 cells after oxalate and IGF1 administration (F). The cells in green fluorescent are in S-phase. The bar plot shows the percentage change of cells in green fluorescent (G). **(H-I)** Western blot staining shows oxalate downregulates, while IGF1 administration upregulates the abundance of p-Akt, p-RB (s780, s795, s807/811), TK1 and Cyclin E1 in HK-2 cells. Two-way ANOVA, Dunnett's multiple comparisons test. *, $p < 0.05$, **, $p < 0.05$; ns, not significant; Scale bar: 50μm
